# Natural VTA activity during NREM sleep influences future exploratory behavior

**DOI:** 10.1016/j.isci.2022.104396

**Published:** 2022-05-13

**Authors:** Julia J. Harris, Mihaly Kollo, Andrew Erskine, Andreas Schaefer, Denis Burdakov

**Affiliations:** 1 System Neuroscience and Energy Control Laboratory, Francis Crick Institute, London, UK; 2 Sensory Circuits and Neurotechnology Laboratory, Francis Crick Institute, London, UK; 3 Department of Neuroscience, Physiology & Pharmacology, University College London, London, UK; 4 Department of Life Sciences, Imperial College London, London, UK; 5 Institute for Neuroscience, Department of Health Sciences and Technology, ETH Zürich, Zürich, CH, Switzerland

**Keywords:** Behavioral neuroscience, Molecular neuroscience, Cellular neuroscience

## Abstract

During wakefulness, the VTA represents the valence of experiences and mediates affective response to the outside world. Recent work revealed that two major VTA populations – dopamine and GABA neurons – are highly active during REM sleep and less active during NREM sleep. Using long-term cell type and brain state-specific recordings, machine learning, and optogenetics, we examined the role that the sleep-activity of these neurons plays in subsequent awake behavior. We found that VTA activity during NREM (but not REM) sleep correlated with exploratory features of the next day’s behavior. Disrupting natural VTA activity during NREM (but not REM) sleep reduced future tendency to explore and increased preferences for familiarity and goal-directed actions, with no direct effect on learning or memory. Our data suggest that, during deep sleep, VTA neurons engage in offline processing, consolidating not memories but affective responses to remembered environments, shaping the way that animals respond to future experiences.

## Highlights

• Dopamine and GABA neurons in the VTA are active during NREM as well as REM sleep
• VTA activity during NREM-sleep — but not REM-sleep — is correlated with exploration the next day
• Inhibiting this activity during NREM-sleep — but not REM-sleep — reduces future exploration

## Graphical abstract

**Figure:**
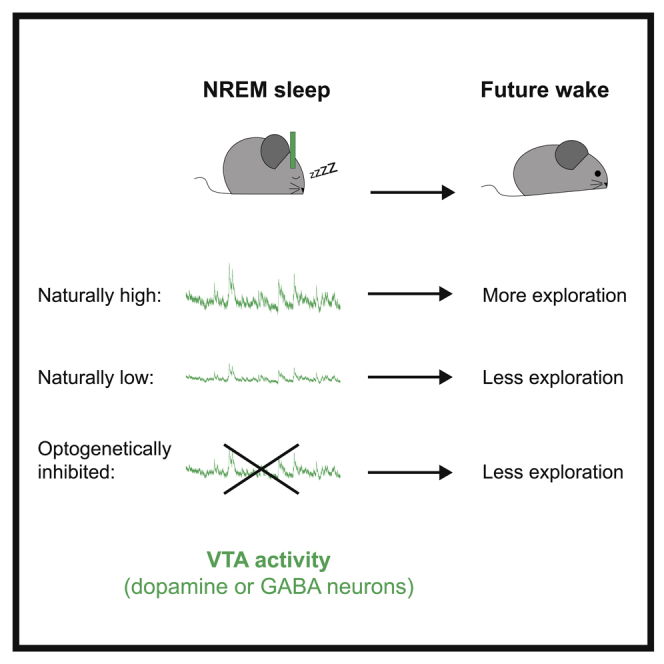

## Introduction

The ventral tegmental area (VTA) is historically implicated in reward processing, reinforcement learning, and affective motivated behavior (Tsai et al., 2009; van Zessen et al., 2012; Mohebi et al., 2019; Dabney et al., 2020; Lee et al., 2020), as well as anxiety/depressive-like phenotypes (Tye et al., 2013; Russo and Nestler, 2013) and exploration and avoidance (Tan et al., 2012). Recently, it was revealed that the VTA also mediates transitions between sleep and wake states. Dopaminergic and glutamatergic neurons in the VTA are necessary for the maintenance of wakefulness (Eban-Rothschild et al., 2016; Oishi et al., 2017; Yu et al., 2019a), whereas VTA GABAergic neurons are necessary for transition to sleep (Yang et al., 2018; Yu et al., 2019a,2019b, Chowdhury et al., 2019).

These three populations of VTA neurons do not act transiently at vigilance state transitions — all of them tend to show low activity during non-rapid eye movement (NREM) sleep and high activity during rapid eye movement (REM) sleep (Eban-Rothschild et al., 2016; Yu et al., 2019a). The function of these sustained differences in activity levels between sleep states is unknown. In the hippocampus and neocortex, neuronal activation patterns are replayed during sleep, likely aiding synaptic reorganization to consolidate wake experiences (Frank and Heller, 2019; Klinzing et al., 2019; Fauth and van Rossum, 2019). Recent studies suggest that VTA dopamine neurons may also be similarly reactivated in quiet rest or sleep after a task is performed (Gomperts et al., 2015; Valdes et al., 2015), and place-cell triggered activation of the medial forebrain bundle during sleep can create a place-field preference in subsequent wakefulness (de Lavilléon et al., 2015). This raises the possibility that the sleep-activity of VTA neurons contributes to offline neuronal processing in a way that shapes future behavior.

To explore this hypothesis, our approach was to first observe the natural activity of VTA neurons during sleep, look for any awake behaviors that correlated with this activity, and then examine how silencing this specific activity during sleep phases affects subsequent awake behaviors. We developed a paradigm in which we could track VTA population activity in mice over a prolonged maze learning experience spanning over four days, including intervening periods of sleep. Using unsupervised time series clustering, we found that exploratory aspects of maze behavior are related to the level of VTA population activity during the previous day’s NREM sleep. We then used targeted sleep-state-specific optogenetic inhibition to selectively disrupt naturally occurring VTA dynamics during REM sleep or NREM sleep periods and analyzed learning, memory, exploration, and novelty preference before and after this manipulation. Importantly, we tailored optogenetic inhibition such that sleep architecture itself remained unperturbed. Because of the large literature implicating a role for sleep in memory (Stickgold, 2005) and the more recent evidence that dopaminergic activity during sleep can influence place preference (de Lavilléon et al., 2015), we expected that any effect of neural disruption during sleep would be most evident in our spatial learning task. Surprisingly, however, we found that inhibiting the natural VTA activity during sleep had no obvious effect on learning or memory. Instead, and in line with our correlation results, VTA inhibition during NREM-sleep — but not REM-sleep — significantly influenced future behaviors relating to exploratory action.

## Results

**Figure 1:**
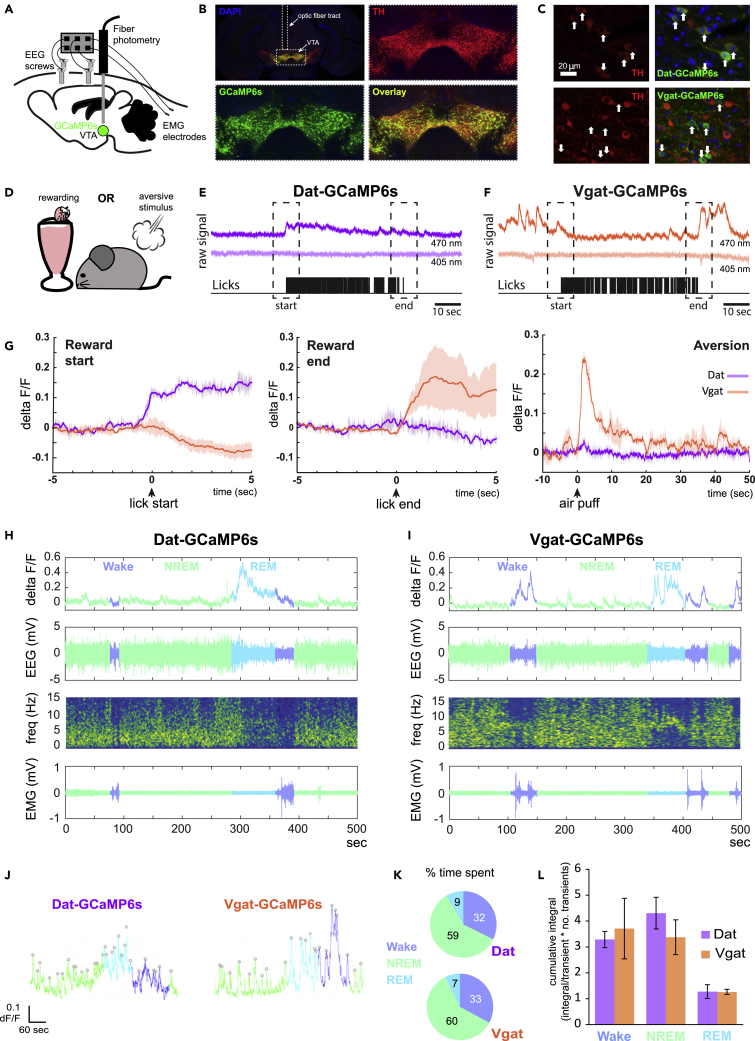
VTA dopaminergic and GABAergic population activity during wake and sleep (A) Schematic of injection site of Cre-dependent GCaMP6s AAV in the VTA of *Vgat-cre* or *Dat-cre* mice. An optic fiber is then implanted directly above the VTA, and EEG screws plus EMG electrodes are implanted and connected to a headstage. (B) Region-specific expression of GCaMP6s in the VTA, where TH-positive dopamine neurons reside. Fiber tract shows position of optic fiber. (C) Cell-specific expression of GCaMP6s. In *Dat-cre* mice (top row), GCaMP6s positive cells (white arrows) co-localize with TH staining. In *Vgat-cre* mice (bottom row), GCaMP6s positive cells do not stain for TH. (D) Task schematic. Mice experience either a rewarding stimulus (free access to strawberry milkshake) or aversive stimulus (air puff to the hind flank). (E) Example recording from VTA^*Dat*^ population during spontaneous milkshake licking. (F) Example recording from VTA^*Vgat*^ population during spontaneous milkshake licking. (G) (Left) Mean responses (plus shaded SEM) across trials from two *Dat-cre* mice and two *Vgat-cre* mice. At the start of milkshake licking, VTA^*Dat*^ population activity increases while VTA^*Vgat*^ population activity decreases. (Middle) The opposite is seen at the end of milkshake licking: VTA^*Dat*^ population activity decreases while VTA^*Vgat*^ population activity increases. (Right) In response to a mildly aversive air puff, VTA^*Vgat*^ population activity transiently increases, whereas VTA^*Dat*^ population activity does not (see Figure S1 for summary statistics). (H) Example photometry, EEG, and EMG recordings from a *Dat-cre* mouse. In the photometry (top row), EEG (second row) and EMG (bottom row) traces, colors represent different stages of sleep. The third row shows the frequencies present in the EEG trace. (I) Example photometry, EEG, and EMG recordings from a *Vgat-cre* mouse. Colors and rows as in H. (J) Example traces showing detection of GCaMP transients in different phases of sleep. (For detection, peak prominence must be > 3xSD of baseline. See STAR Methods for details.) (K) The average amount of time spent in different vigilance states over the first 4 h of rest in a new light cycle. For both *Dat-cre* and *Vgat-cre* mice, the majority of time is spent in NREM sleep, whereas the least amount of time is spent in REM sleep (2 *Dat-cre* mice and 4 *Vgat-cre* mice). (L) VTA neurons are more active during REM sleep than NREM sleep, but they are not silent during NREM sleep. Summing the activity over different vigilance states (i.e., taking the cumulative integral of activity transients in each state) reveals that — over a 4 h rest period — the total NREM activity VTA neurons of VTA^*Dat*^ and VTA^*Vgat*^ populations tends to be greater than the total REM activity, significantly so for *Vgat-cre* mice (paired *t*-test between NREM and REM means: p = 0.036; n = 4) but not for *Dat-cre mice* (paired *t*-test between NREM and REM means: p = 0.072, n = 2). Means are plotted ± SEM.

Photometry recordings of genetically defined VTA populations expressing GCaMP6s during wake (Figures 1A–1G) showed that dopaminergic (DAT-expressing, VTA^*Dat*^) neurons increased their activity during a rewarding event (self-paced milkshake licking, Figures 1D,1E, 1G, and S1A). In contrast, GABAergic (VGAT-expressing, VTA^*Vgat*^) neurons decreased their activity during the same event (Figure 1D, 1F, 1G, and S1A). When an aversive air puff was given, a near-opposite pattern was seen: VTA^*Vgat*^ neurons increased their activity, whereas VTA^*Dat*^ neurons did not respond (Figures 1G and S1A).

During sleep, however, the same two populations may behave similarly; both dopaminergic and GABAergic neurons in the VTA have been reported to show decreased activity during NREM sleep as compared to wake and REM sleep (Eban-Rothschild et al., 2016; Yu et al., 2019a). Because different subpopulations of VTA neurons can encode different aspects of behavior (Lammel et al., 2011, 2012; Cohen et al., 2012; Morales and Margolis, 2017), we wanted to examine whether this similarity in sleep activity held true for the very same neuronal populations in which we observed opposing wake activity. Thus, we carried out sleep recordings in the same mice that had received rewarding and aversive stimuli during wake. We found that both VTA^*Dat*^ and VTA^*Vgat*^ populations showed decreased activity during NREM sleep as compared to wake and REM sleep (Figures 1H and 1I), supporting the idea that these VTA populations are similarly activated during sleep despite responding very differently to specific wake-delivered stimuli.

The vigilance-related changes in natural VTA activity happen almost immediately upon transition between states and are sustained until the next state change: both neuronal populations showed decreased activity on the transition from wake to NREM sleep, increased activity on the transition from NREM to REM sleep, no obvious change on the transition from REM sleep to wake, and increased activity on the transition from NREM sleep to wake (Figure S1B). Both populations tended to have more transients during REM sleep as compared to NREM sleep (Figures 1J and S1C and S1D), and the size of individual transients may be slightly larger during REM sleep than NREM sleep (Figures S1C and S1E), but neither population was entirely silent during NREM sleep. Besides, because mice spend much more time in NREM than REM sleep (Figures 1K and S1F), the total NREM activity during a sleep episode — measured as the population activity summed over time — tends to be greater than the total REM activity for both populations (of course the exact difference will depend on the total length of the rest period: here, it was 4 h; Figure 1L). This suggests that the activity during NREM sleep could in fact play a significant role in neural processing.

**Figure 2:**
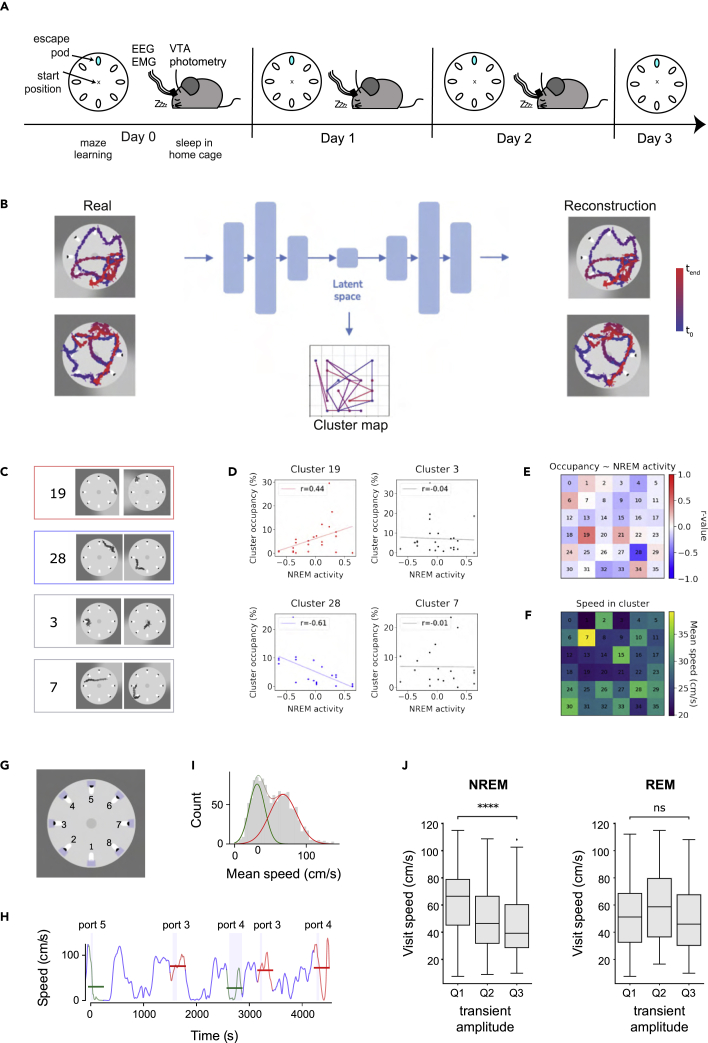
Relationship between natural VTA activity during sleep and the next day’s behavior (A) Schematic of the recording paradigm that produced the data entered into the variational autoencoder. Each day for a period of four consecutive days, mice undertook five maze learning trials, followed by a 4 h period of sleep in their home cage; during this time, photometry was used to measure VTA population activity during different sleep states. We investigated the relationship between sleep photometry from one day and behavioral trajectories in the maze the next day (represented by the red arrows). (B) Schematic of the variational autoencoder model used to cluster behaviors, consisting of an encoder, which reduces the input (left panel, a set of behavioral variables from tracking 10 s segments of a real mouse in the Barnes maze) into four latent features, and a decoder, which generates samples from the latent space (producing a virtual mouse which reproduces the behavior of the real mouse, right panel). The model projects the continuous trajectories in the latent space onto a self-organizing map (SOM), converting the mouse behavior into a series of transitions between behavior clusters (“cluster map”). The color code indicates trial time (blue: beginning of trial, red: end of trial). (C) Example behaviors from four behavioral clusters: one positively correlated with NREM sleep (red frame), one negatively correlated (blue frame), and two uncorrelated (gray frames). (D) Scatterplots showing the correlation between the VTA population activity (standardized transient amplitude, see STAR Methods) during NREM and relative time spent (“occupancy”) in 4 example clusters during behavioral tasks on the following day. (The r values for Pearson’s correlation are indicated. Red indicates a positive correlation, blue indicates a negative correlation, and gray indicates no correlation). (E) Heatmap showing the Pearson’s correlation coefficient of the cluster occupancy and VTA population activity during the preceding NREM sleep periods for each cluster. (F) Heatmap showing the average speed of the mouse during exploration for each cluster. (G) Definition of port zones (blue shaded areas) and port numbers in the Barnes maze. (H) Speed of the mouse during exploration of the maze. Port visits (vertical, blue shaded areas, number of port are indicated above) were sometimes accompanied with deceleration and low average speed (green line) and other times with high speed (red line). Horizontal green and red lines indicate the mean speed around the start of the visit (−0.5 s–2.5 s from the start of entering the port area). (I) The mean speed during port visits is bimodally distributed. The green and red curves indicate the two Gaussian components describing the slow and fast type port visits. (J) Port visits are faster on days following NREM sleep with small GCaMP transients compared to days with high GCaMP transients. Differences in REM activity are not associated with following day port-visit behavior. (MWU-test between the lower and upper quartiles, ∗∗∗∗: p ≤ 1.00x10-4, Boxes indicate IQR, midline indicates median).

If average activity is higher during REM sleep, but cumulative activity is higher during NREM sleep, which phase of activity — if either — is relevant for future behavior? To investigate this question, we developed a paradigm in which we could track spontaneous VTA population activity over a prolonged period of maze learning, with intervening periods of sleep for four days in a row (Figure 2A). Each day, the mice explored a modified Barnes maze (see STAR Methods), in which they had to use spatial cues to learn which sheltered escape pod provided escape from a bright lit circular arena. Mice were then allowed to rest and sleep in their home cage; during this time, we measured VTA population activity across sleep-wake states (defined by simultaneous EEG/EMG recordings). We then used a machine learning approach to identify specific maze behaviors and examine whether any of these behaviors were related to the observed VTA activity during the preceding sleep period.

The behavior of animals in complex environments, while highly dynamic, shows strong modularity (Wiltschko et al., 2015). In simple organisms, behavioral motifs can be readily identified with dimensionality reduction techniques (Stephens et al., 2008; Berman et al., 2014), but the complex and long dynamics of mouse movement in a free-form maze are more challenging. We decided to use deep unsupervised learning to cluster mouse behavior in an unbiased way. Specifically, we employed a variational autoencoder architecture (Rezende et al., 2014; Kingma and Welling, 2014), which consists of two parts: an encoder, which reduces the input into a limited set of latent features, and a decoder, which generates samples from this latent space. The latent representations learned by these models are relatively disentangled, meaning that each latent unit is sensitive to individual generative factors of the input data (Kingma and Welling, 2014).

In our analysis, we used a previously published and tested model (TempDPSOM, Manduchi et al., 2020), which performs the encoding and clustering of time series simultaneously. We trained the network to encode and reliably reconstruct 10 s snippets of mouse movement through the maze, described by coordinates of the head, center, and tail of the animal and the total area occupied by the mouse (Figure 2B). Using the encoder network, we successfully compressed each time series (consisting of 100 time steps and seven features) into a latent vector of 4 dimensions, from which the decoder network could reliably reconstruct movement trajectories (Figure 2B). The model was simultaneously optimized to project the latent space onto a discrete self-organizing map (SOM), where the animal gets assigned to one of 36 behavioral clusters at every time point (Figures 2B and 2C). Each cluster contained variable behaviors, but stereotypical motifs could be identified in individual clusters (Figure S2).

We then examined whether the proportion of time that mice spent in each behavioral cluster was correlated with the level of natural VTA activity during their preceding sleep period. We found that two of the 36 clusters were highly correlated with prior NREM activity (Figures 2D and 2E). Interestingly, both of these clusters included port-investigation behaviors, but one cluster was positively correlated with NREM activity (cluster 19), whereas the other was negatively correlated (cluster 28). Closer analysis of these clusters revealed that the positively correlated cluster was more often associated with long investigations of ports, whereas the negatively correlated cluster frequently contained brief port visits in which the animal made a fast approach and departure. Notably, clusters associated with other high-speed behaviors were not correlated with NREM activity (Figures 2F and S2), indicating that NREM activity did not have a simple, general relationship with the following day’s speed but was specifically related to the rapidity of port investigation.

This result guided us to create a simple metric from the raw behavioral data: port visit speed. Specifically, we calculated the mean speed of the mouse before (0.5 s) and after (2.5 s) each entry into a port zone (Figure 2G, see STAR Methods for full details). This metric showed a bimodal distribution, representing two types of port visits: extensive slow visits and fast pass-and-go visits (Figures 2H and 2I). We found that port visit speed was negatively correlated with VTA activity during preceding NREM sleep periods but was not correlated at all with VTA activity during the preceding REM period (Figure 2J). These results suggest that naturally high VTA activity during deep sleep may promote deliberate, investigative behaviors the following day, whereas naturally low VTA activity during deep sleep may suppress future exploration.

**Figure 3:**
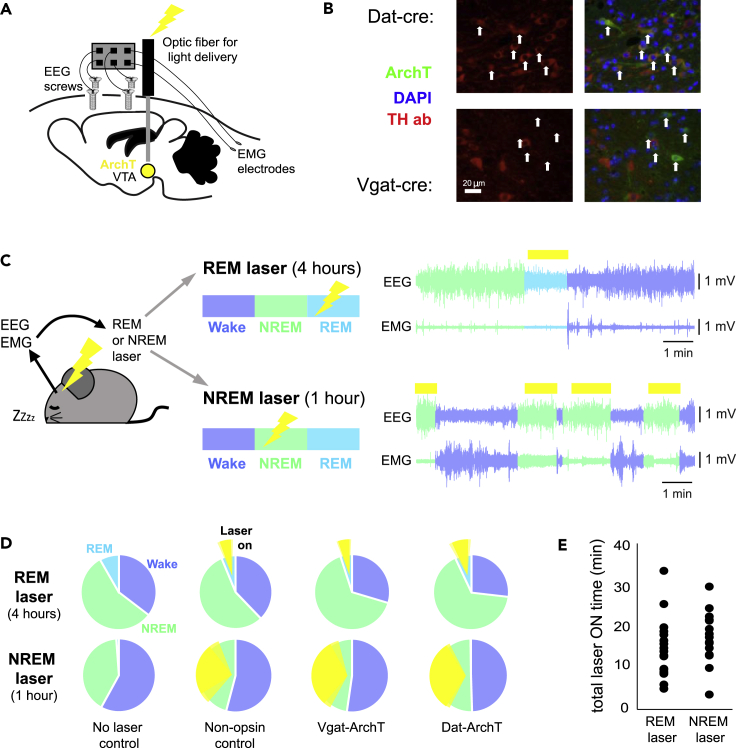
State-specific optical silencing of dopaminergic and GABAergic VTA populations (A) Schematic of injection site of cre-dependent ArchT AAV in the VTA of *Vgat-cre* or *Dat-cre* mice. An optic fiber is then implanted directly above the VTA, through which excitation light (532 nm) is delivered. EEG screws plus EMG electrodes are implanted and connected to a headstage. (B) Cell-specific expression of ArchT. In *Dat-cre* mice (top row), ArchT positive cells (white arrows) co-localize with TH staining. In *Vgat-cre* mice (bottom row), ArchT positive cells do not stain for TH. (C) Schematic of light delivery protocols for REM laser and NREM laser experimental conditions. EEG and EMG signals are continuously monitored online. In the REM laser condition, 532 nm light is delivered during episodes of REM sleep (top row). In the NREM laser condition, 532 nm light is delivered during episodes of NREM sleep (bottom row). (D) Average proportion of time spent in each sleep state across all mice in each experimental condition (REM laser: no laser control n = 8, non-opsin control n = 5, *Vgat-cre*-ArchT n = 6, *Dat-cre*-ArchT n = 6; NREM laser: no laser control n = 5, non-opsin control n = 5, *Vgat-cre*-ArchT n = 7, *Dat-cre*-ArchT n = 6). Yellow represents the average laser on time in each condition (SEM in light yellow). (E) The total laser ON time during NREM experiments (1 h) or REM experiments (4 h) is not significantly different (Student’s *t* test not significant, p > 0.1).

To test this hypothesis causally, we experimentally inhibited the naturally occurring VTA activity during either NREM or REM sleep and examined the effects on future behavior. Specifically, we targeted the optogenetic inhibitory actuator ArchT or a non-opsin control protein (see STAR Methods) to the dopaminergic or GABAergic neurons of the VTA (Figures 3A and 3B) and used an implanted light fiber to optically inhibit these populations during either REM sleep (where activity is high but bouts are few) or NREM sleep (where activity is low but highly cumulative over time) (Figures 3C and 3D). The same surgeries, injections, and laser procedures were applied in the control groups, where the non-opsin protein meant that neural activity was not manipulated. State-specific optical manipulation was carried out during a rest period between tasks, where the mouse was returned to its home cage and allowed to sleep freely. This rest period was 4 h for the REM laser condition, because REM episodes do not typically occur during the first 2 h of sleep, but by 4 h, REM sleep is present, and the distribution across different arousal states is similar to what is seen across a full 12 h light phase (Figure S3A, cf. Soltani et al., 2019 their Figure 2). To avoid a difference in total silencing time, the rest period for the NREM laser condition was only 1 h as the first hour of the day’s rest is naturally split between wake and NREM episodes only (the distribution of arousal states across the NREM experiment matched the first hour of the REM experiment, Figure S3A). This created an equivalence in the total laser ON time between experimental conditions (Figure 3E).

**Figure 4:**
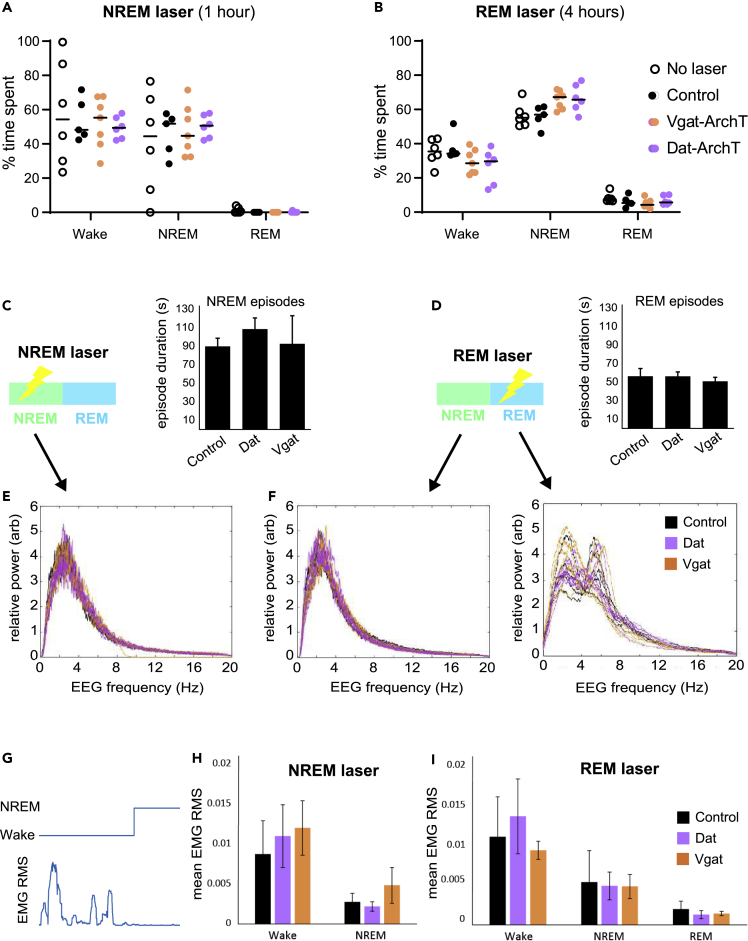
Sleep was not disrupted by state-specific opto-silencing in the VTA (A) Light delivery during NREM sleep did not affect the amount of time spent in different vigilance states for *Vgat-cre*-ArchT (orange) or *Dat-cre*-ArchT (purple) mice (student’s *t*-tests did not reveal any significant difference between groups for any vigilance state). (B) Light delivery during REM sleep did not affect the amount of time spent in different vigilance states for *Vgat-cre*-ArchT (orange) or *Dat-cre*-ArchT (purple) mice (student’s *t*-tests did not reveal any significant difference between groups for any vigilance state). (C) VTA inhibition during NREM sleep did not alter NREM episode length (Student’s *t*-tests: control vs Dat p = 0.26; control vs Vgat p = 0.94; n numbers as in B). (D) VTA inhibition during REM sleep did not alter either REM episode length (Student’s *t*-tests: control vs Dat p = 0.99; control vs Vgat p = 0.54; n numbers as in B). (E) FFT representing the power across the frequencies present during NREM sleep episodes (expressed relative to the total NREM power) for each mouse plotted individually. In this condition, the laser was ON during these NREM episodes, but a two-way repeated measures ANOVA did not reveal a difference in the spectral profiles between mouse groups (F(2,12) = 0.23, p = 0.23; control n = 5, Dat n = 4, Vgat n = 6). (F) FFTs representing the relative power across all frequencies present during NREM sleep episodes (left) and REM sleep episodes (right) for each mouse plotted individually. In this condition, the laser was ON during REM episodes. Two-way repeated measures ANOVAs did not reveal a difference in the spectral profiles between mouse groups for either NREM episodes (F(2,15) = 3.63, p = 0.052) or REM episodes (F(2,15) = 0.18, p = 0.83; control n = 5, Dat n = 6, Vgat n = 7). (G) Example trace of EMG root-mean-square (RMS, bottom) plotted against arousal state (top). (H) No significant differences were found between experimental and control groups in either Wake (control vs Dat p = 0.70; control vs Vgat p = 0.55) or NREM episodes (control vs Dat p = 0.65; control vs Vgat p = 0.48) during the NREM laser condition (Student’s *t*-tests, control n = 5, Dat n = 6, Vgat n = 7). (I) No significant differences were found between experimental and control groups in Wake (control vs Dat p = 0.72; control vs Vgat p = 0.73), NREM (control vs Dat p = 0.91; control vs Vgat p = 0.89) or REM (control vs Dat p = 0.54; control vs Vgat p = 0.58) episodes during the REM laser condition. (Tests and n numbers as in H). For all bar graphs, means are plotted ±SEM.

Because it has been shown that VTA populations play a role in transitions between sleep and wakefulness, we wanted to ensure that our optogenetic approach did not directly disrupt sleep architecture; therefore, we avoided optogenetic activation because previous work has demonstrated that for both the dopaminergic (Eban-Rothschild et al., 2016) and GABAergic (Yu et al., 2019a,b) populations, this causes sleep state transitions within seconds of stimulation. With our unilateral opto-inhibition approach, we found that state-specific optical manipulation did not alter the time spent in each arousal state, compared to wild-type no-laser controls or non-opsin controls, which received the same laser treatment (Figures 4A and 4B). Turning the laser on during REM or NREM episodes neither did alter the length of these episodes (Figures 4C and 4D) nor did affect the total number of state transitions (Figure S3B). The EEG spectral profiles of NREM and REM sleep were not altered when the laser was turned on during these episodes (Figures 4E and 4F right) nor were there any apparent nonspecific alterations to NREM sleep when the laser was turned on during REM sleep (Figure 4F left; note the opposite comparison could not be made as there was typically no REM sleep in the 1 h NREM condition). Finally, opto-silencing did not alter EMG indicators of arousal in any state (Figures 4G–4I). However, acute opto-inhibition during wake, did affect consummatory licking behavior similarly to what has been previously described for Vgat (Van Zessen et al., 2012) and Dat (Hughes et al., 2020; Lee et al., 2020) neurons (Figure S4). In addition, *chronic* opto-inhibition of each population during rest (laser on continuously for 4 h, beginning in quiet wake) did alter sleep architecture in drastically opposing ways, consistent with chemogenetic and sustained opto-manipulation results from previous studies (Eban-Rothschild et al., 2016; Yu et al., 2019a,b; Figure S5).

**Figure 5:**
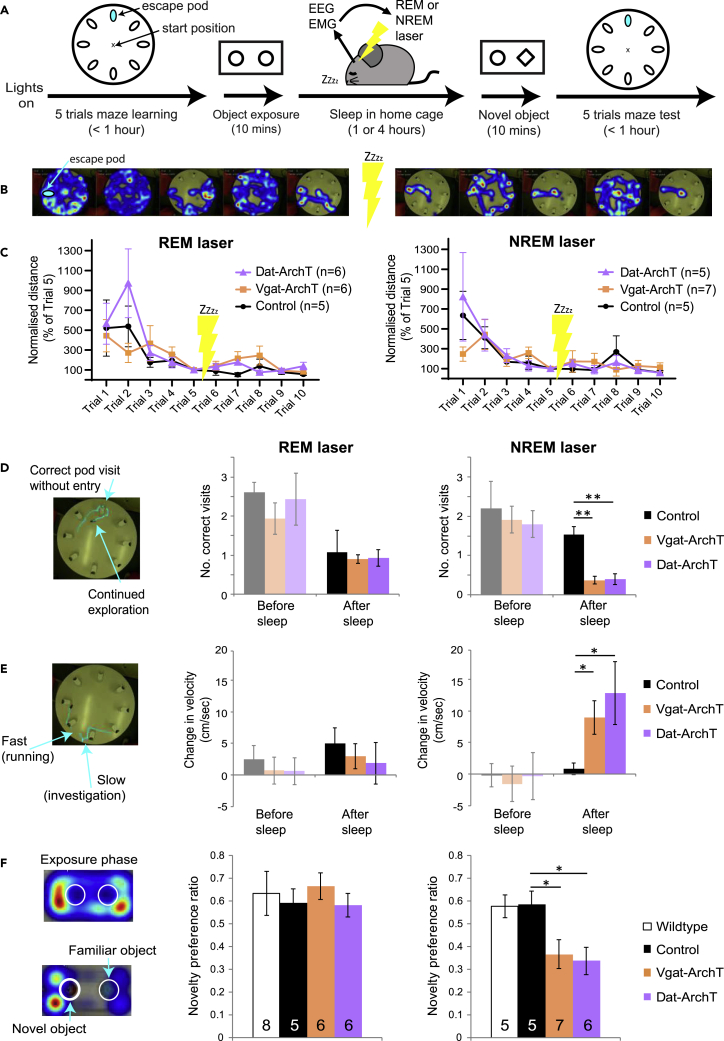
VTA silencing during sleep affects future tendency to explore but does not disrupt learning (A) Sequence of behavioral testing. At lights on (when sleep pressure is high), the experiment starts with 5 trials in a Barnes maze, followed by habituation to two identical novel objects. The mouse is then returned to its home cage and allowed to rest and sleep freely, for one or four hours. During this time, arousal state is continuously monitored using EEG and EMG, and laser light is delivered either during bouts of REM sleep or NREM sleep (as in Figure 3C). The mouse is then exposed to one habituated object and one novel object and is finally placed in the Barnes maze for 5 more trials. (B) Example behavior (spatial occupancy color map) of one mouse in the maze (non-opsin control mouse, with laser on during REM). In this case, the correct escape pod is at 9 O’clock. (C) Distance traveled in each trial before escape normalized to the distance traveled on the final trial before sleep. For all mice, the distance traveled decreases significantly with trial number, showing that they have all learned the task (two-way repeated measures ANOVA: REM: main effect of trial, p = 0.0004, no interaction effects; NREM: main effect of trial, p = 0.002, no interaction effects). Disrupting VTA population activity during REM or NREM sleep does not disrupt learning of this task. (D) After sleep, mice tend to make fewer visits to the correct pod, but control mice do sometimes visit the correct pod without entering, in favor of exploring the maze a little more fast (example tracking, left panel). Silencing VTA^*Dat*^ or VTA^*Vgat*^ populations during REM sleep does not affect this behavior (middle panel). However, silencing either population during NREM sleep significantly reduces the number of correct pod visits after sleep, compared to control (right panel; student’s *t*-test: *Vgat-cre*-ArchT: p < 0.01; *Dat-cre*-ArchT: p < 0.01). In other words, these mice are more likely to enter the correct pod the first time they come across it. (E). As learning starts to progress, mice tend to increase their average velocity in the maze compared to the first trial, but they also pause to sniff and investigate their surroundings (example tracking, left panel). Silencing VTA^*Dat*^ or VTA^*Vgat*^ populations during REM sleep does not alter this behavior (middle panel). However, silencing either population during NREM sleep significantly augments the post-sleep velocity increase compared to control mice (right panel; student’s *t*-test: *Vgat-cre*-ArchT: p < 0.05; *Dat-cre*-ArchT: p < 0.05). (F) When mice are exposed to two identical objects, they spend a similar amount of time exploring each object. When one of these objects is replaced with a novel object, they typically spend more time exploring the novel object (example tracking, left panel). Wild-type mice (no laser) and non-opsin control mice display this behavior, spending approximately 60% of their object-exploration time with the novel object. Silencing VTA^*Dat*^ or VTA^*Vgat*^ populations during REM sleep does not affect this behavior (middle panel). However, after silencing either population during NREM sleep, mice switch to preferring the familiar object (only approximately 35% of their object-exploration time is spent with the novel object). This behavior is significantly different from non-opsin controls (right panel; t-test: *Vgat-cre*-ArchT: p = 0.038; *Dat-cre*-ArchT: p = 0.018). For all bar graphs, means are plotted ±SEM.

To investigate the behavioral effects of the sleep-state-specific VTA –inhibition, which importantly did not interfere with sleep itself (Figures 4 and S3), we used two learning and memory paradigms. The first was the modified Barnes maze to look at spatial learning, and the second was a novel object paradigm to look at object recollection. The experiment began with lights on, when mice would normally have high sleep pressure. Each mouse performed five trials in the Barnes maze, followed by phase 1 of the object test (first exposure to two identical objects; Figure 5A). The mouse was then returned to its home cage for a rest period of one or 4 h while the laser was turned on during NREM or REM sleep, respectively. Immediately after sleep, mice performed phase 2 of the object test, where a novel object replaced one of the familiar objects. Mice then completed five more trials in the Barnes maze. Over these ten trials in the maze, mice tended to find the escape pod with less maze coverage and shorter path lengths (Figure 5B), indicating spatial learning. Inhibiting VTA^*Dat*^ or VTA^*Vgat*^ populations during REM or NREM sleep did not impair maze learning as measured by the distance traveled before correct pod entry (Figure 5C). We were initially surprised by this result, as abundant literature suggests that sleep is crucial for learning and memory. However, as our manipulation did not interfere with sleep amount or architecture and was not directed at canonical memory centers of the brain, perhaps memory effects were not, in fact, to be expected. Instead, closer analysis of mouse behavior in the maze revealed that VTA interference during NREM sleep significantly affected the way in which the animals interacted with the remembered maze environment.

After sleep, mice tended to make fewer visits to the correct pod, but control mice did sometimes visit the correct pod without entering in favor of exploring the maze a little more fast (Figure 5D, left panel). Inhibiting VTA^*Dat*^ or VTA^*Vgat*^ populations during REM sleep did not affect this exploration behavior (middle panel). However, inhibiting either population during NREM significantly reduced the number of correct pod visits after sleep, compared to control (right panel; Student’s *t* test: VTA^*Vgat*^: p < 0.01; VTA^*Dat*^: p < 0.01). In other words, these mice were more likely to enter the correct pod the first time they came across it and less likely to continue exploring the maze “unnecessarily.” As learning progressed, mice tended to increase their average velocity in the maze compared to the first trial, but they also paused to investigate their surroundings (Figure 5E, left panel). Inhibiting VTA^*Dat*^ or VTA^*Vgat*^ populations during REM sleep did not alter this behavior (middle panel). However, inhibiting either population during NREM sleep significantly augmented the post-sleep velocity increase, compared to control mice (right panel; Student’s *t*-test: VTA^*Vgat*^: p < 0.05; VTA^*Dat*^: p < 0.05). Importantly, the ArchT groups did not differ from control groups before manipulation, and control groups did not differ from each other either before or after manipulation: Figure S6. In addition, we did not find any general effects on arousal, locomotion, or anxiety after manipulation: Figures S7 and S8. Thus, the activity of these neurons during sleep does not appear critical to spatial learning or general levels of arousal/anxiety, but disrupting these neurons during NREM sleep does affect future behavior, seeming to reduce behaviors that favor exploration and investigation and promote goal-directed action.

To further examine this association between NREM VTA intrinsic activity and investigative drive, we employed the classic novelty preference paradigm in combination with state-specific optogenetic silencing. When mice are exposed to two identical objects, they spend a similar amount of time exploring each object. When one of these objects is replaced with a novel object, they typically spend more time exploring the novel object (Figure 5F, left panel). Wild-type mice (no laser) and non-opsin control mice displayed this behavior, spending approximately 60% of their exploration time with the novel object (Figure 5F, middle and right panels). Inhibiting VTA^*Dat*^ or VTA^*Vgat*^ populations during REM sleep did not affect this behavior (middle panel). However, after inhibiting either population during NREM sleep, mice switched to preferring the familiar object (right panel: only approximately 35% of their exploration time is spent with the novel object). This behavior was significantly different from controls (student’s t-tests: VTA^*Vgat*^: p = 0.038; VTA^*Dat*^: p = 0.018). Similar to the effects of optogenetic inhibition in the Barnes maze, this result does not suggest an effect on memory itself; i.e., if memory were eradicated, then mice would spend 50% of their time with each object (as in the exposure phase). Instead, it seems that the mice remember the objects but prefer to spend time with the familiar object rather than exploring the new object, again suggesting that natural VTA activity during NREM sleep is linked to future exploratory drive. Collectively, these photomanipulation results support the picture that VTA activity during NREM sleep is linked to exploratory and investigative behaviors in subsequent wakefulness.

## Discussion

We have found that naturally occurring VTA activity during NREM sleep is important for shaping future exploratory behavior. Analysis of VTA intrinsic activity and behavioral measures that were tracked over several days suggested that high natural VTA activity during NREM — but not REM — sleep is related to prolonged investigation in subsequent wakefulness. Complementarily, inhibiting normal VTA activity during NREM — but not REM — sleep reduced the future drive for novelty and exploration, in favor of familiarity and goal-directed escape responses.

### NREM vs REM

Because endogenous VTA activity is low in NREM sleep compared to REM sleep, the possible importance of VTA neuron activation during NREM sleep has been overlooked. Our results demonstrate that — in fact — the natural VTA activity that is present in NREM sleep is important for future behavior. We were initially surprised to find that our machine learning analysis did not indicate a substantial relationship between endogenous REM VTA activity and behavior the following day. Similarly, inhibiting VTA neurons during REM sleep had no observable effect on future behavior. On the other hand, NREM sleep is where the majority of replay events occur (outside of quiet wakefulness: O’Neill et al., 2010; Atherton et al., 2015; Klinzing et al., 2019), and our data therefore align with the idea that endogenous VTA activity during NREM sleep may contribute to the offline processing of wake experiences. Interestingly, the VTA does not appear to influence memory consolidation during this time but rather modulates the way in which an animal will react to a remembered object or environment in the future. Specifically, NREM VTA activity appears to promote future investigative behaviors, whereas the absence of this natural activity limits future exploratory drive, making animals respond to their environment in a more reserved manner with a preference for familiarity.

### Dopamine vs GABA

Another unexpected result was that despite the two major populations in the VTA — dopaminergic and GABAergic — behaving oppositely during wake (Figure 1), the behavioral effects of inhibiting either population during sleep was indistinguishable. Thus, VTA populations which respond oppositely during wakefulness appear to behave synergistically in sleep. Previous work also shows that the three VTA populations (dopaminergic, GABAergic, and glutamatergic) covary their activity during sleep, all becoming quieter during NREM bouts and highly active during REM bouts (Eban-Rothschild et al., 2016; Yu et al., 2019a). More recently, Eban-Rothschild et al. (2020) found that the VTA dopaminergic and GABAergic populations were differentially correlated with key EEG power bands during wake but similarly correlated during NREM sleep. It is possible that sleep propels the VTA into a different activity regime not coupled to ongoing behavior, with the effect of temporally separating neural processing from action as proposed for other brain areas (Kaufman et al., 2014). Interestingly, strong silencing of these populations (optogenetically: our Figure S5; Chowdhury et al., 2019; and chemogenetically: Eban-Rothschild et al., 2016; Yu et al., 2019a,b) does lead to different effects on sleep architecture, suggesting perhaps that these populations are differentially connected with sleep/wake centers of the brain (e.g., the LH: Yu et al., 2019a). Disrupting sleep architecture itself makes it impossible to examine the contribution of these different populations on future behavior, which is why we kept our optogenetic manipulation below the threshold of sleep disruption. What is clear from the present data is that interfering with either population alters the way that the VTA normally influences offline tuning of behavioral responses to remembered environments, even when sleep architecture remains intact.

### Arousal and motivated behavior

An animal’s need for sleep must be balanced with potential benefits of awake-motivated behavior, such as exploring a new environment for food or mates (Sotelo et al., 2020). Dopaminergic neurons in the VTA are involved in this trade-off: silencing them during wake prevents animals from having an appropriate arousal response to salient and motivating features in their environment and instead promotes sleep-preparatory behaviors and sleep (Eban-Rothschild et al., 2016). Sleep itself has been proposed to restore an animal’s readiness to perform goal-directed rather than habit-driven behaviors (Vyazovskiy et al., 2017), and our work suggests that the VTA might play a role in this: silencing natural VTA activity during deep sleep promotes a preference for familiarity over novelty/investigation in the next waking episode. Thus, over both wake and sleep, the VTA could help mediate the complex trade-off between exploring salient features of an environment versus taking advantage of familiar features that guarantee safety and perhaps allow for rest.

### Memory and anxiety

Sleep is considered to play a key role in learning and memory, and poor sleep can also have profoundly negative emotional consequences. Therefore, we were initially surprised to see no direct effects on learning (maze learning trajectories were not affected, Figure 5C; and the memory for novel objects was not eradicated, Figure 5F) nor any general effects on anxiety (Figure S7). However, a key feature of this particular study is that sleep itself is not disrupted, and therefore we did not eliminate all of the neuronal processing that normally occurs during sleep. Instead, by manipulating specific neuronal populations without disrupting sleep itself, we have been able to reveal a distinct role for the activity of a subset of neurons during sleep. Specifically, in the absence of normal VTA activity during deep sleep, animals responded differently to recently experienced environments — they explored less and preferred familiar objects, even though general anxiety levels were unaffected. We are all aware that one benefit of a good night of sleep is that stressful things seem more manageable the next day; although yesterday’s stresses are not forgotten, they simply feel less scary to approach. Speculatively, we believe it is this role of sleep that we have hit upon here; i.e., natural VTA activity during deep sleep may process the affective aspects of recent salient experiences in such a way that can shift future behavior toward a calmer and more curious response to remembered situations.

### Limitations of the study

We would like to point out two key limitations of the present study. First, the behaviors that we could identify were of course limited by the tasks that we gave the mice to perform. It is possible that other behaviors are even more strongly linked to VTA activity during deep sleep and even that entirely different behaviors are linked to VTA activity during REM sleep. It will be interesting to repeat these experiments with a wide variety of behavioral tests (e.g., reward conditioning or social tasks). Second, although we have shown a bidirectional correlation between natural VTA activity during sleep and future behavior, our causal evidence is unidirectional. We purposefully did not attempt a “sufficiency” experiment in this study for two reasons. First, optogenetic VTA stimulation is unlikely to be possible without waking the animal up (Yu et al., 2019a; Eban-Rothschild et al., 2016). Second, during sleep, neurons are known to be reactivated in highly specific spatiotemporal patterns, often in a sparse manner (Lewis and Bendor, 2020). As we cannot currently predict this activity, there is no reason to be confident that artificial opto-stimulation could mimic its natural occurrence in any meaningful way.

We would also like to point out a caveat against comparing our NREM and REM conditions. The rest period allowed for the NREM condition was 1 h, whereas that allowed for the REM condition was 4 h. This experimental design is a response to the natural phenomenon that the day’s sleep starts with NREM, and REM episodes do not appear until two-three hours later. The occurrence of these phases and their latency from sleep onset will therefore always be inextricably linked, and there is no way to decouple this relationship without introducing great confounds. For this reason, we included control groups within each condition which experience the exact same timing, and the appropriate comparisons are between experimental groups and within-condition controls. It is crucial to bear in mind that the NREM and REM conditions should not be directly compared to each other, and that the conclusions drawn in one condition are independent of those drawn in the other.

### Broader implications

During wake, the VTA has been known to be important for representing the valence of experiences and mediating our affective responses to the outside world. Our data now suggest that during deep sleep, VTA neurons continue to engage in neural processing, consolidating not memories but affective responses to remembered environments and ultimately shaping the way in which animals respond to future experiences. Because of the known links between sleep and mood disorders (reviewed in Wulff et al., 2010; Ben Simon et al., 2020), our results have implications for mental health. For example, manipulating deep sleep VTA activity using translational tools such as noninvasive brain stimulation (Polania et al., 2018) may offer a new opportunity for therapeutic treatment of affective disorders. More importantly, although NREM sleep occupies up to a quarter of life in mammals (Colten and Altevogt, 2006), its function remains incompletely understood. By revealing that natural VTA activity during NREM sleep is linked to future awake behavior, our findings illuminate a new function of NREM sleep.

## STAR★Methods

### Key resources table

**Table:** 

REAGENT or RESOURCE	SOURCE	IDENTIFIER
**Antibodies**		

rabbit polyclonal TH	Chemicon	AB152 Sigma-Aldrich
rabbit polyclonal Vgat	Synaptic Systems	Cat. No.: 131,013
goat polyclonal GFP	Abcam	ab6673
Alexa Fluor 488 anti-goat	Invitrogen	Catalog # A32814
Alexa Fluor 555 anti-rabbit	Invitrogen	Catalog # A-21428

**Bacterial and virus strains**		

rAAV9.CAG.Flex.GCaMP6s.WPRE.SV40	UPenn Vector Core	Addgene cat no: 100,842
rAAV8/Flex-ArchT-GFP	UNC GTC Vector Core	Addgene cat no: 28,307

**Deposited data**		

Natural VTA activity during NREM sleep influences future exploratory behavior	Mendeley Data	https://doi.org/10.17632/mffncst8fw.1
mkollo/VTA_NREM	Github	https://github.com/mkollo/VTA_NREM

**Experimental models: Organisms/strains**		

Dat-cre	Jackson Laboratories; Zhuang et al. (2005)	RRID:IMSR_JAX:020,080
Vgat-ires-cre	Jackson Laboratories; B. Lowell	RRID:IMSR_JAX:016,962

**Software and algorithms**		

MATLAB	MATLAB R2020b	https://www.mathworks.com/
Python	Python Software Foundation	https://www.python.org
Spike2	Version 8	https://ced.co.uk/products/spkovin

### Resource availability

#### Lead contact

Further information and requests for resources and reagents should be directed to and will be fulfilled by the lead contact, Julia Harris (julia.harris@crick.ac.uk).

#### Materials availability

This study did not generate new unique reagents.

### Experimental model and subject details

#### Animals

Animal research has been approved by United Kingdom Home Office and the Animal Welfare and Ethical Review Panel of the Francis Crick Institute. All procedures were carried out in accordance with the Animals (Scientific Procedure) Act of 1986. Mice were kept on a standard 12-h/12-h light/dark cycle on standard mouse chow and water *ad libitum*. Adult mice (at least 14-weeks old) were used for experiments. In accordance to NC3Rs practice (https://www.nc3rs.org.uk/the-3rs), both males and females were used for this study. Behavioral experiments were performed during the first hours of the light phase.

### Method details

#### Genetic targeting

Two previously characterized mouse lines were used expressing Cre recombinase in DAT-expressing or VGAT-expressing neurons: *Dat-cre* (knock-in; Zhuang et al., 2005) and *Vgat-ires-cre* (transgenic from Jackson Laboratories and B. Lowell, Harvard University Fukunaga et al., 2014). To target expression of the calcium indicator, GCaMP6s or the optical silencer, ArchT to either VTA^*Dat*^ or VTA^*Vgat*^ neurons, we injected a cre-dependent AAV9.CAG.Flex.GCaMP6s.WPRE.SV40 (titer: 2.74x10e13 vg/ml; UPenn Vector Core) or AAV8/Flex-ArchT-GFP (titer: 4.6x10e12 vg/ml; UNC GTC Vector Core) into the VTA of *Dat-cre* or *Vgat-cre* mice.

GCaMP6s was used as a calcium indicator for the photometry experiments, or as a non-opsin control for the optogenetics experiments (as in our other studies: Kosse and Burdakov, 2019; Concetti and Burdakov, 2021). While YFP/GFP are more traditionally used as non-opsin control molecules, we find that GCaMP is a good non-toxic alternative (there are reports that YFP and GFP can be toxic; YFP: Comley et al., 2011; GFP: Ansari et al., 2016), which otherwise is functionally the same in its use as a non-opsin control under the opto-inhibitory laser regime. Additionally, this strategy makes the same mice that are used as controls in the sleep/behavior experiments available for awake recordings of neural activity (e.g. response to rewarding/aversive stimuli, Figures 1D–1G). As well as providing extra assurance that GCaMP is expressed in the correct cells, this maximises the experimental use of single animals, which is vitally important for raising ethical standards.

#### Surgeries

For brain surgeries, mice were anesthetized with isoflurane and injected s.c. with meloxicam (2 mg/kg of body weight) for analgesia. After positioning in a stereotaxic frame (Kopf Instruments), a craniotomy was performed and borosilicate glass pipette was used to inject viral vectors unilaterally into the VTA. Two injections (100 nL each) were made into the VTA at the following coordinates from bregma: AP -3.4, ML +/−0.48, depth 1: −4.4, depth 2: −4.3. After injections, mice were implanted with four miniature screw electrodes (from bregma: AP +1.5 and ML +1.5 (ground); AP +1.5 and ML -1.5 (common reference); AP -1.5 and ML -1.5 (EEG 1); AP -1.5 and ML +1.5 (EEG 2) and two EMG electrodes (inserted into neck musculature). These electrodes were each connected, via an insulated wire, to a different gold pin of a EEG/EMG headstage. A fiberoptic implant (200 um diameter) was stereotactically installed with the fiber tip above the VAT (AP -3.4, ML +/− 0.48, depth of tip: −4.1). This method is estimated to capture fluorescence signals from within approximately 500 um of the fiber tip (González et al., 2016). The EEG/EMG headstage and external portion of the fiberoptic cannula were affixed to the skull using dental adhesive resin cement (Super-Bond C&B). Mice were allowed to recover for at least ten days before handling, and experiments were performed from 2 weeks (for GCaMP6s) up to four months (for ArchT) post-surgery. Littermates were kept together post-surgery. Optic fiber and viral placements are shown in Figure S9.

#### Fiber photometry

During fiber photometry experiments, pulses of 470 nm excitation light were interleaved with pulses of 405 nm light via LEDs (M470F3 and M405FP1; Thorlabs), alternating at 20 Hz. Fluorescence emission produced by 405 nm excitation is calcium-independent and thus provides a real-time control for motion artifacts (Kim et al., 2016). Light power was between 70 and 100 uW, kept constant for each mouse. Emitted photons (≈525 nm) were captured by a photodetector (PDF10A Femtowatt receiver, Thorlabs) and data was recorded using Spike2 via a CED Micro1401-3 data acquisition unit (Cambridge Electronics Design) at a sampling rate of 400 Hz. Fluorescence signals were normalized as follows: ΔF/F = (F_r_-F_m_)/F_m_, where F_r_ is the raw signal and F_m_ is the median of either the 10 seconds before a stimulus (for reward and aversion experiments) or the entire photometry recording (for maze and sleep experiments). For sleep experiments, where the photometry signal was recorded for up to four hours in one session, there was a slight decrease in the baseline signal over time. To de-trend the baseline, a simple polynomial was fitted and subtracted before calculating ΔF/F, as above. For transient classification (Figure 1J and 1L), a 250 second “quiet period” was manually identified during NREM sleep, for each trace. The threshold for initial peak detection was set to 3 times the SD of this quiet period. Peaks with a half-width shorter than 1.5 seconds were filtered out, and all remaining peaks were plotted for visual confirmation.

#### EEG and EMG recordings and vigilance state classification

EEG and EMG signals were recorded using the Pinnacle 3-channel tethered system (8200-K1-SL; Pinnacle Technology Inc). Signals were filtered by the preamplifier (high pass above 0.5 Hz for EEG and above 10 Hz for EMG) and then recorded in Spike2, via the CED box. Sleep states – NREM, REM and wake – were automatically classified using sleep analysis software in Spike2, and then manually verified in 5 second epochs. Wakefulness was defined as de-synchronised, low amplitude EEG and tonic EMG with bursts of movement. NREM sleep was defined as synchronized, high amplitude EEG in the delta frequency range (1-4 Hz) and reduced EMG activity relative to wakefulness. REM sleep was defined when EEG had reduced delta power but prominent power in the theta range (4-10 Hz), and EMG showed an absence of muscle tone.

#### Chronic and state-specific optical inhibition

For optical silencing experiments, a green laser (532 nm, LaserGlow Technologies) was connected to the fibre implant to yield ≈20 mW light power output at the fiber tip. For chronic inhibition, the laser was turned on when the animal was awake (in their home cage), and kept on continuously for four hours. For state-specific inhibition, mice were allowed to sleep in their home cage while an experimenter continuously monitored EEG and EMG activity. The real-time vigilance state was determined based on the criteria above. For the REM laser condition, EEG and EMG activity was monitored for four hours and, whenever the mouse entered REM sleep, the green laser was manually activated and light was continuously delivered to the VTA via the implanted optical fiber until the mouse transitioned out of REM sleep. The laser was then turned off until the next REM sleep episode occurred. Individual REM episodes were rarely longer than two minutes. For the NREM laser condition, EEG and EMG activity was monitored for one hour and, whenever the mouse entered NREM sleep, the laser was manually activated. If the episode of NREM sleep lasted longer than two minutes, the laser was turned off for 5 seconds to minimize the unwanted side effects of any heat damage by the laser, and then turned on again. This was repeated until the mouse transitioned out of NREM sleep. The laser was then turned off until the next NREM sleep episode occurred. Optical inhibition experiments were blinded: the experimenter was blind to the genotype of the mouse at the time of the experiment itself (for the majority of experiments) and for EEG and behavior analysis (for all experiments).

#### Rewarding and aversive stimuli

Strawberry milkshake (Frijj) was used as a rewarding stimulus. To habituate the mice to the milkshake, the night before the experiment, food was removed from the cage and milkshake was provided in addition to water. For the experiment, mice were placed singly in a small arena with free access to milkshake from a water bottle. A lick sensor recorded each lick, and photometry signals were acquired simultaneously, both recorded in Spike2. Mice remained in the arena for 10 minutes from the first lick. For the aversion experiment, mice were placed singly in a home-cage-like arena, and a ∼500 ms air puff was delivered to the base of the tail once a minute, three to five times per mouse.

#### Barnes maze

We created a modified Barnes maze so that it could be used in conjunction with tethered fiber photometry. We devised “escape pods” instead of holes, so that the mice could enter a comforting space while still having the optical fiber attached. The pods were painted white externally to aid video tracking, but were black on the inside as mice prefer dark spaces. Before the start of the experiment, mice were habituated to the escape pods by placing one in their home cage. Mice were considered habituated when a hand entering the cage caused the mice to take refuge inside the pod.

For the experiment, 8 escape pods were arranged evenly around the outside of a circular arena (120 cm in diameter and 115 cm above the floor). Seven pods had closed doors, and only one pod with an open door could be entered. The pods were positioned with their doors facing away from the center of the arena, such that a mouse could only see which pod was accessible when directly looking at it from the outer edge of the arena. Mice began each trial by being placed in a well below the center of the maze. To disorient the mice and remove memory of room spatial cues, the mice were kept in the well for 30 seconds at the start of each trial. The well platform was then raised by a programmed motor to be level with the arena, so mice entered the maze at its center, facing a random direction. From here, mice were able to use spatial cues around the room to navigate to the correct escape pod. These include visual cues (e.g. checkerboard pattern) and olfactory cues (e.g. home cage and experimenter), which were kept in the same place in the room for each trial. When the mice found and entered the correct pod, the entire pod was immediately placed into their home cage (acting like a teleportation device to home). If the correct pod was not found within 10 minutes, the mouse was gently picked up and placed in front of the correct pod. Once back in the home cage, the mice were allowed a 2-5 minute inter-trial break. During this time, intra-maze olfactory cues were eradicated by careful cleaning of the arena and pods, and individual pods were shuffled into different positions. The open pod was kept in the same location for each mouse, but the identity of the pod itself was shuffled for each trial.

The open pod was assigned to different locations for different mice. In the four-day photometry experiment, the mice performed five maze trials per day. In the optical inhibition experiment, the mice performed a total of ten maze trials in a single day (separated by a period of sleep). NREM laser and REM laser conditions were completed by the same mice, using a counterbalanced design: half of the mice performed the NREM laser condition first and the other half performed the REM laser condition first. There was at least a one-week gap between conditions, and the location of the open pod was changed. Because we wanted to study post-learning sleep, experiments were started within the first two hours of the light cycle (between 7 and 9 am).

Videos of the mice in the maze were captured using a Raspberry Pi camera mounted on the ceiling above the maze, and synchronized to the physiological recordings via frame-by-frame TTL pulses sent to Spike2 (via CED box; 10 frames per second). Nose, center and tail tracking was then performed using Ethovision software. We calculated position in the maze, cumulative distance, and instantaneous velocity for each frame of each trial. We also examined number of visits to the correct pod, which was defined as the mouse nose entering a 3 cm perimeter around the correct pod. If, rather than entering the pod, the mouse exited this perimeter or turned their nose away from the pod, this was classified as a correct pod visit without entry (Figure 5D).

#### Novel object

For the first phase of the novel object experiment, mice were placed in a familiar arena with two identical novel objects for ten minutes. The mice were then returned to their home cage for one or four hours, where they were allowed to sleep. Directly afterwards, mice were returned to the experimental arena, which now contained one object from the first phase (familiar object) and one brand new object (novel object). They were allowed to explore freely for ten minutes. Videos were captured using a Raspberry Pi camera, and mice were tracked using Ethovision. An animal was considered to be exploring an object when its nose was within a 2 cm perimeter around the object. We calculated a novelty preference ratio as follows: time_novel_/time_novel_ + time_familiar_, where time_novel_ is the time spent exploring the novel object, and time_familiar_ is the time spent exploring the familiar object.

Choice of objects (from four in total) for familiar and novel phases were counterbalanced between mice and between optical inhibition conditions to avoid any confounding factors due to differences in objects. To keep the timing between tasks consistent (which is essential for assessing memory), the novel object task (which was of fixed length - 10 minutes), was always flanked by the Barnes maze task (which was of variable length according to mouse performance - 13-60 minutes).

#### Immunohistochemistry

50 um fixed slices were stained using the following primary antibodies: rabbit polyclonal TH (1:500; Chemicon) or rabbit polyclonal Vgat (1:1000; Synaptic Systems) and goat polyclonal GFP (1:300; Abcam). Secondaries were Alexa Fluor 488 anti-goat (Molecular Probes) and Alexa Fluor 555 anti-rabbit (Invitrogen). Slices were DAPI-stained and mounted on slides, and images were captured using a Nikon NIS microscope or a Zeiss Axioscan slide scanner.

### Quantification and statistical analysis

All error bars and shaded regions represent +/−- SEM. Where multiple t-tests or Mann-Whitney U tests are used, p values are adjusted using Bonferroni correction for multiple comparisons.

#### Deep variational autoencoder model for maze behavior

We utilized a deep architecture for probabilistic time-series clustering (Temp-DPSOM, Manduchi et al., 2021) combining a variational autoencoder (Kingma and Welling, 2014; Rezende et al., 2014), forecasting in the latent space using LSTMs and a self-organizing map (SOM) for clustering input time-series samples. Behavioral trials were described in a 7 dimensional time series sampled at 10 Hz, where the first 6 dimensions corresponded to the maze-center-referenced X and Y coordinates of the center of the body, the relative positions of the nose-end and tail-end of the body, and the area of the animal. The input to the VAE network was prepared by creating overlapping samples from the trajectories from all trials with a 10 second timing window, resulting in samples of 100 time-steps and 7 features. The model was trained on the training dataset (85% of samples, batch size of 100, 256 epochs), and validated on 15% of the samples from a different set of trials. For training the model, behavioral trajectories of 5 trials on each of 4 days were used from 6 animals (4 *Vgat-cre* and 2 *Dat-cre*). Training was finished after no further improvement of reconstruction accuracy was found on the validation dataset. After training the VAE model, each 10 second sample of behavior from the 4 *Vgat-cre* mice on 3 trial days, starting with the second trial day, was converted into a series of transitions in the cluster-space made up of 36 behavioral clusters.

#### Correlation of sleep fiber photometry and awake behavior

To correlate behaviour with fluorescence signals measured during sleep, for each mouse, day and trial, the ratio of time spent in each of the 36 clusters (occupancy) was calculated. Fiber photometry values were standardized between animals, and detrended across days. Pearson’s correlation with occupancy values for each mouse, day and trial was calculated.

#### Quantification of port visits

Port zones were defined as the area between 50-58 cm from the maze centre, within 6 degree deviation from the entrance of each port. Port entry was defined as any time the nose of the mouse entered into one of these zones. Port visit speed was defined as the mean absolute speed of the nose of the mouse from −0.5 to 2.5 s from port entry.

## Data Availability

The data presented in this paper have been deposited on Mendeley Data and are publicly available as of the date of publication. DOIs are listed in the key resources table. All original code has been deposited on Github and is publicly available as of the date of publication. DOIs are listed in the key resources table. Any additional information required to reanalyze the data reported in this paper is available from the lead contact upon request.
