# Supplementary material for: Natural VTA activity during NREM sleep influences future exploratory behavior
Source: iScience. 2022 May 13;25(6):104396. doi: 10.1016/j.isci.2022.104396 (PMC9156940; doi:10.1016/j.isci.2022.104396)
Supplement: Document S1. Figures S1–S9 [file mmc1.pdf]

iScience, Volume *25*

Supplemental information

Natural VTA activity during NREM sleep influences future exploratory behavior

Julia J. Harris, Mihaly Kollo, Andrew Erskine, Andreas Schaefer, and Denis Burdakov

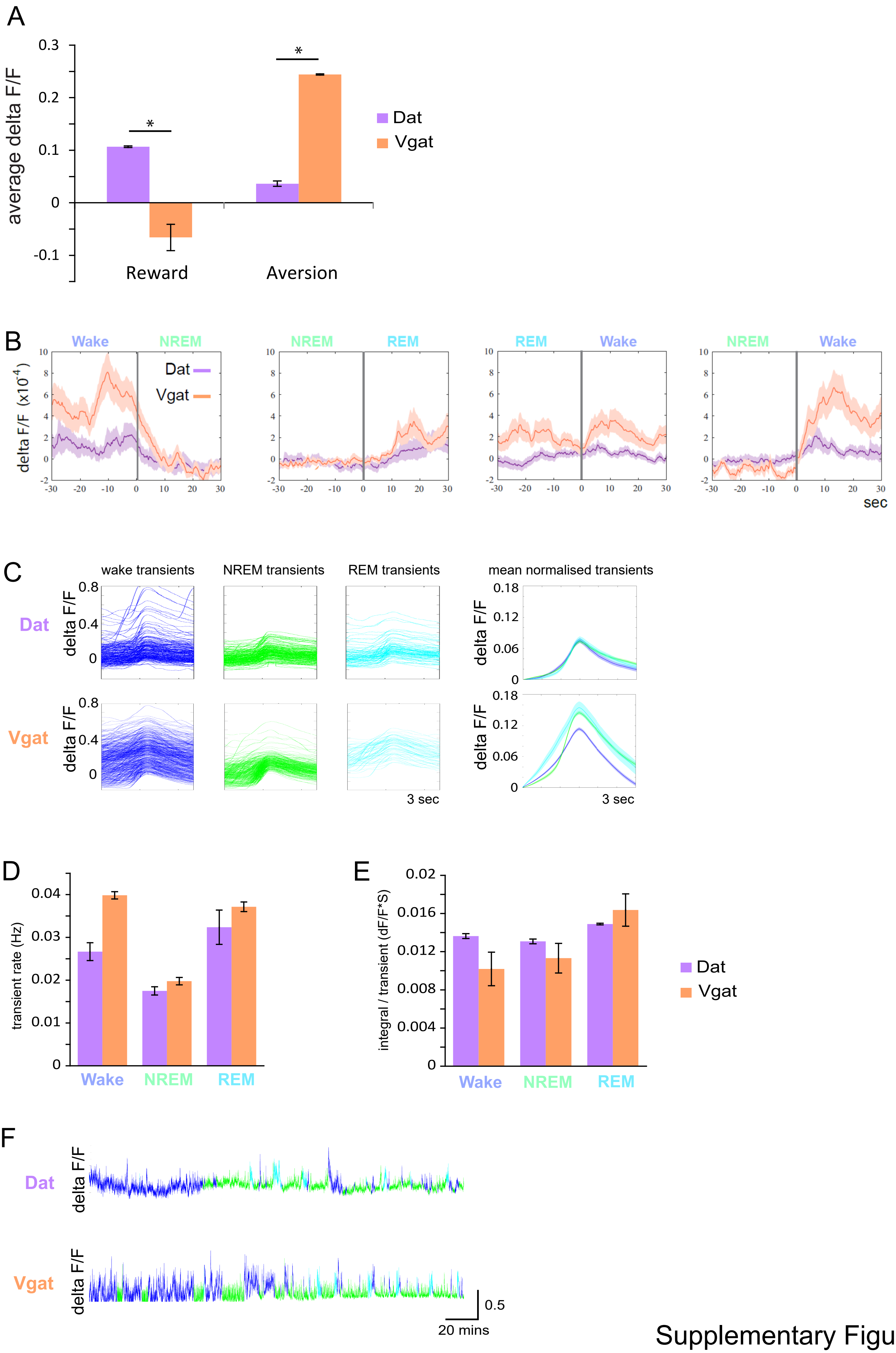

Supplementary Figure 1

Figure S1: Wake and sleep photometry measures (related to Figure 1)

A. VTA dopaminergic and GABAergic populations respond significantly differently to rewarding stimuli (student's t-test, p=0.020) and aversive stimuli (student's t-test, p < 0.001). The bar graphs show, for reward, the average deltaF/F across the duration of licking and, for aversion, the average deltaF/F at the peak response after the air puff (2 *Dat-cre* mice and 2 *Vgat-cre* mice). Error bars represent +/- S.E.M.

B. VTA^{Dat} and VTA^{Vgat} population responses to state transitions, averaged across all transitions in a single period of sleep, for example mice. Unlike the opposing responses to wake experiences (Figure 1), VTA^{Dat} and VTA^{Vgat} populations follow the same trend at each state transition: activity in both decreases from wake to NREM (*Vgat-cre* mouse transitions n=21; *Dat-cre* mouse transitions n=24), increases from NREM to REM (*Vgat-cre* mouse transitions n=11; *Dat-cre* mouse transitions n=9), shows no clear change from REM to wake (*Vgat-cre* mouse transitions n=11; *Dat-cre* mouse transitions n=9), and increases from NREM to wake (*Vgat-cre* mouse transitions n=9; *Dat-cre* mouse transitions n=15).

C. For an example *Dat-cre* and *Vgat-cre* mouse, all of the transients identified during wake, NREM and REM are shown. The individual traces demonstrate that wake and REM transients often ride on top of a higher baseline level of fluorescence, whereas NREM transients tend to start from a near-zero fluorescence signal. The graphs on the right show these transients normalized to the value at the start of each transient.

D. Photometry transient rate was typically higher in REM sleep than NREM sleep. This was significant for our *Vgat-cre* (p < 0.001, n=4) but not our *Dat-cre* (p = 0.13, n=2) group (paired t-tests).

E. The average transient integral appeared slightly higher in REM sleep compared to NREM sleep, but this was not significant for either *Vgat-cre* (p = 0.051, n=4) or *Dat-cre* (p = 0.11, n=2) mice (paired t-tests).

F. Example photometry trace from a *Dat-cre* and a *Vgat-cre* mouse over the first four hours of rest in a new light cycle.

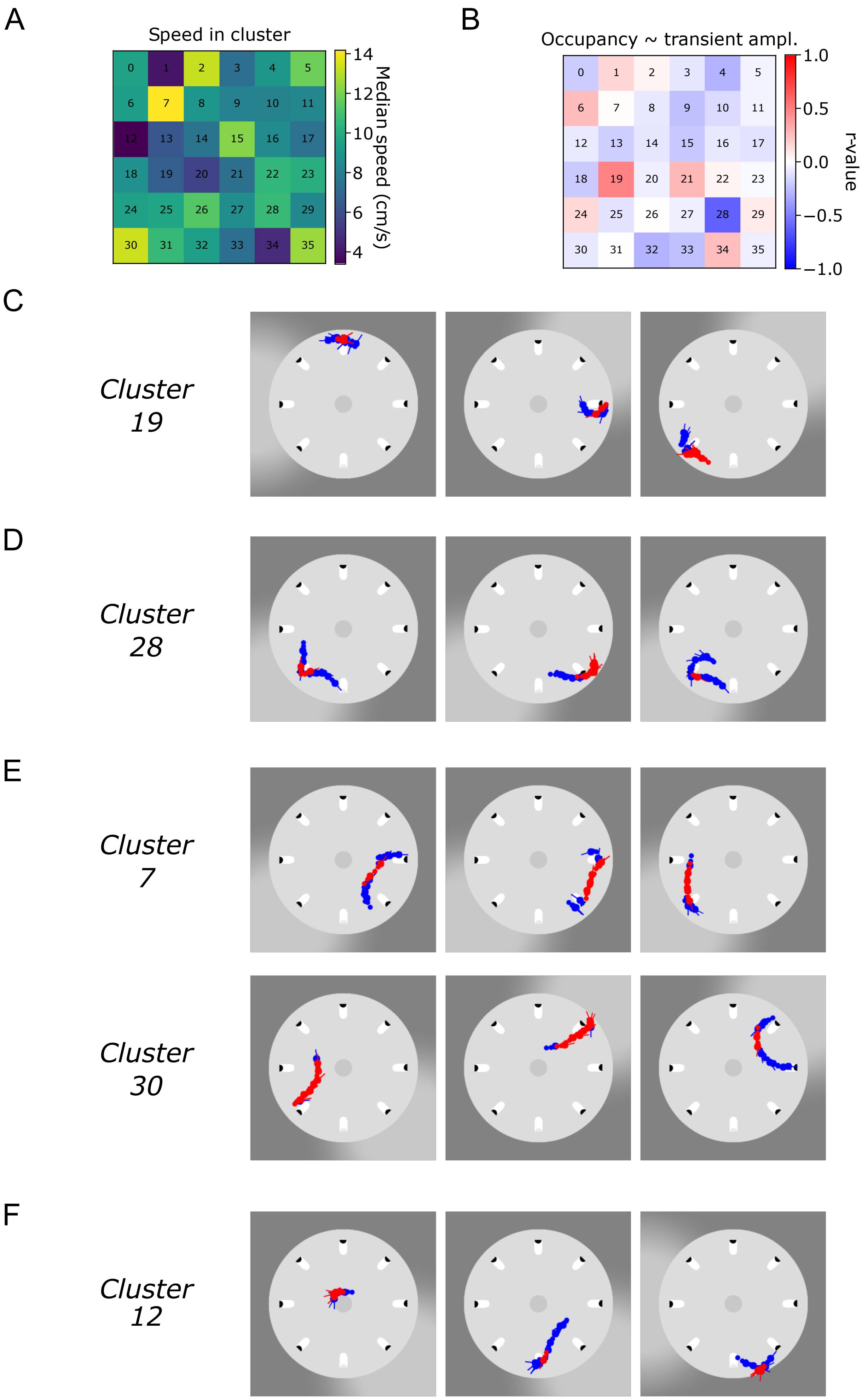

Supplementary Figure 2

Figure S2: Behavioural clusters (related to Figure 2)

A. Heatmap showing the median speed of the mouse while performing behaviours in each behavioural cluster.

B. Heatmap showing the Pearson's correlation coefficient of the relative amount of time that VGAT-GCaMP mice spend each day in different clusters (occupancy), and VTA population activity (standardized transient amplitude) during the preceding NREM sleep periods.

C. Example behaviours from cluster 19, the cluster most positively correlated with NREM activity, displaying extensive investigation during port visits. ±1.5 seconds around the occurrence of the cluster is shown. Red mice indicate when the mouse was assigned to cluster 19, blue mice are drawn when the behaviour was assigned to different clusters. Every 5th sample is shown for display purposes.

D. Example behaviours from cluster 28, the cluster most negatively correlated with NREM activity. The cluster contains many brief, pass-and-go visits to ports.

E. Example behaviours from clusters with high average speed, but not correlated with NREM activity. Clusters 7 and 30 include high-speed running through the central area of the maze and between ports.

F. Example behaviour from low-average-speed cluster 19, not correlated with NREM, which contains many brief stops during movement in areas away from ports.

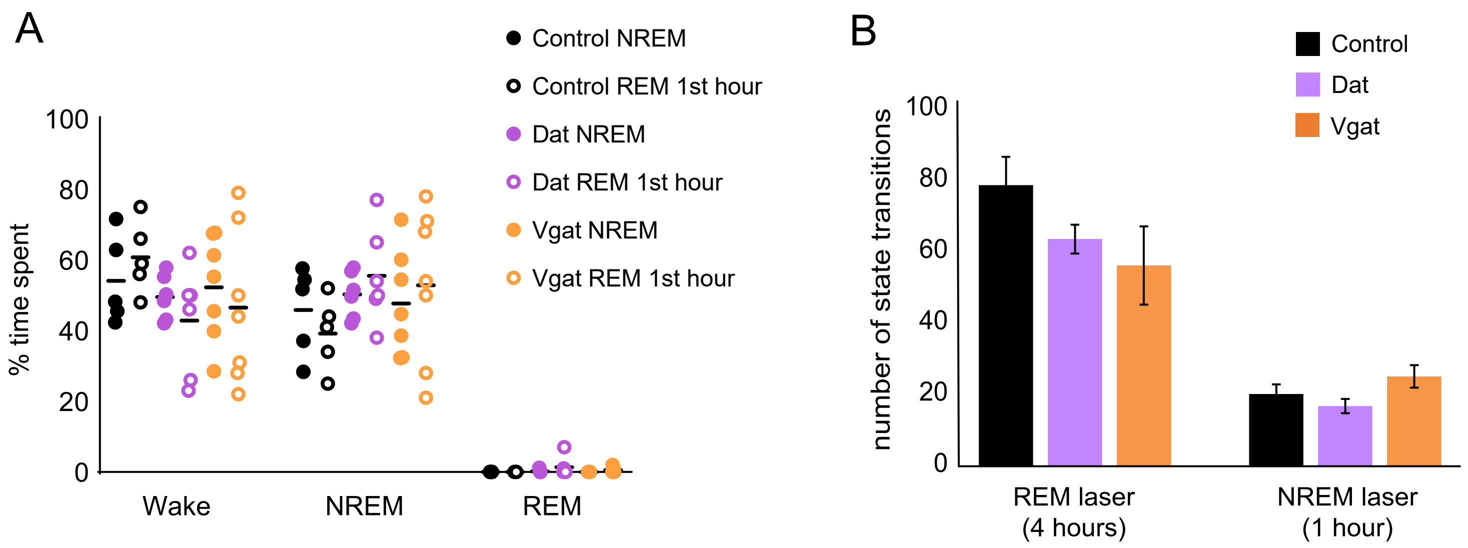

Supplementary Figure 3

Figure S3: Sleep state distributions and transitions (related to Figures 3 and 4)

A. The % time spent in each arousal state was similar between the whole hour of the NREM laser condition (closed circles) and the first hour of the REM laser condition (open circles), for each experimental group (each circle represents one mouse).

B. The number of state transitions was not significantly different between experimental groups for either the REM laser (control vs Dat: p=0.11; control vs Vgat: p=0.16) or the NREM laser condition (control vs Dat: p=0.34; control vs Vgat: p=0.29). (Student's t-tests, control n=5; Dat n=6; Vgat n=7).

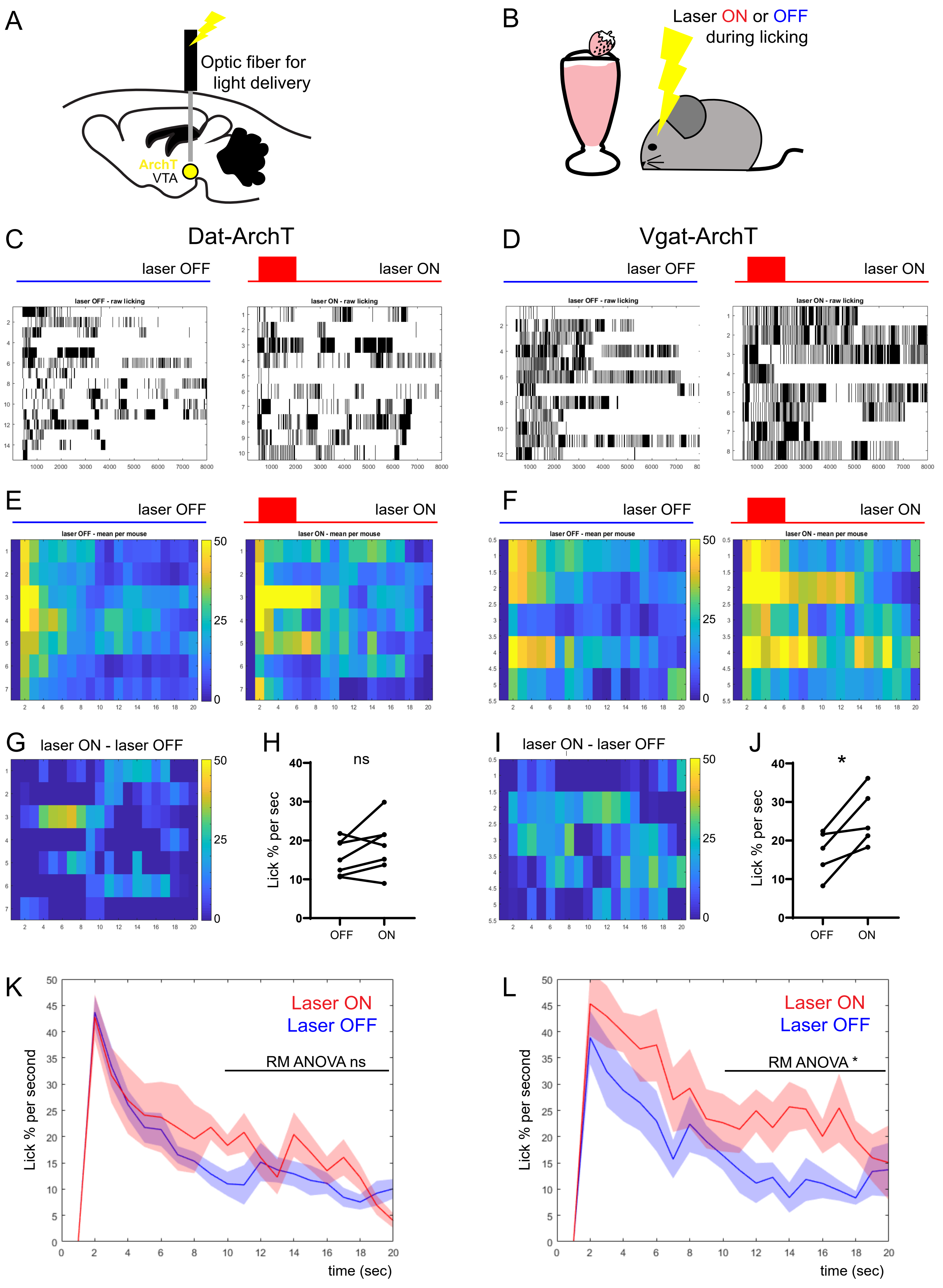

Supplementary Figure 4

Figure S4: Opto-inhibition during consummatory licking (related to Figure 3)

A. Experimental set-up for delivering light to activate cre-dependent ArchT in the VTA.

B. In this experiment, animals were allowed to freely lick a milkshake. The laser was alternately turned ON for 5 seconds or left OFF during licking bouts.

C. Example lick traces for all laser OFF and laser ON licking bouts for an example Dat-ArchT mouse.

D. Example lick traces for all laser OFF and laser ON licking bouts for an example Vgat-ArchT mouse.

E. Average licks per second per mouse during milkshake licking bouts either without any neuronal interference (left) or with 5 second laser activation of ArchT expressed in dopaminergic VTA neurons (right).

F. Average licks per second per mouse during milkshake licking bouts either without any neuronal interference (left) or with 5 second laser activation of ArchT expressed in GABAergic VTA neurons (right).

G. Visualisation of the difference between lick rate for Laser ON vs Laser OFF trials for each Dat-ArchT mouse.

H. Total lick rate was not affected by dopaminergic inhibition (paired t-test; $p = 0.15$).

I. Visualisation of the difference between lick rate for Laser ON vs Laser OFF trials for each Vgat-ArchT mouse.

J. Total lick rate was increased by GABAergic inhibition (paired t-test; $p = 0.022$).

K. For the Dat-ArchT group, a two-way repeated measures ANOVA did not reveal a difference in licking after the laser was turned off ($F (1.000, 6.000) = 2.055$, $P=0.2017$).

L. For the Vgat-ArchT group, a two-way repeated ANOVA did reveal a difference in licking after the laser was turned off ($F (1.000, 4.000) = 13.87$, $P=0.0204$).

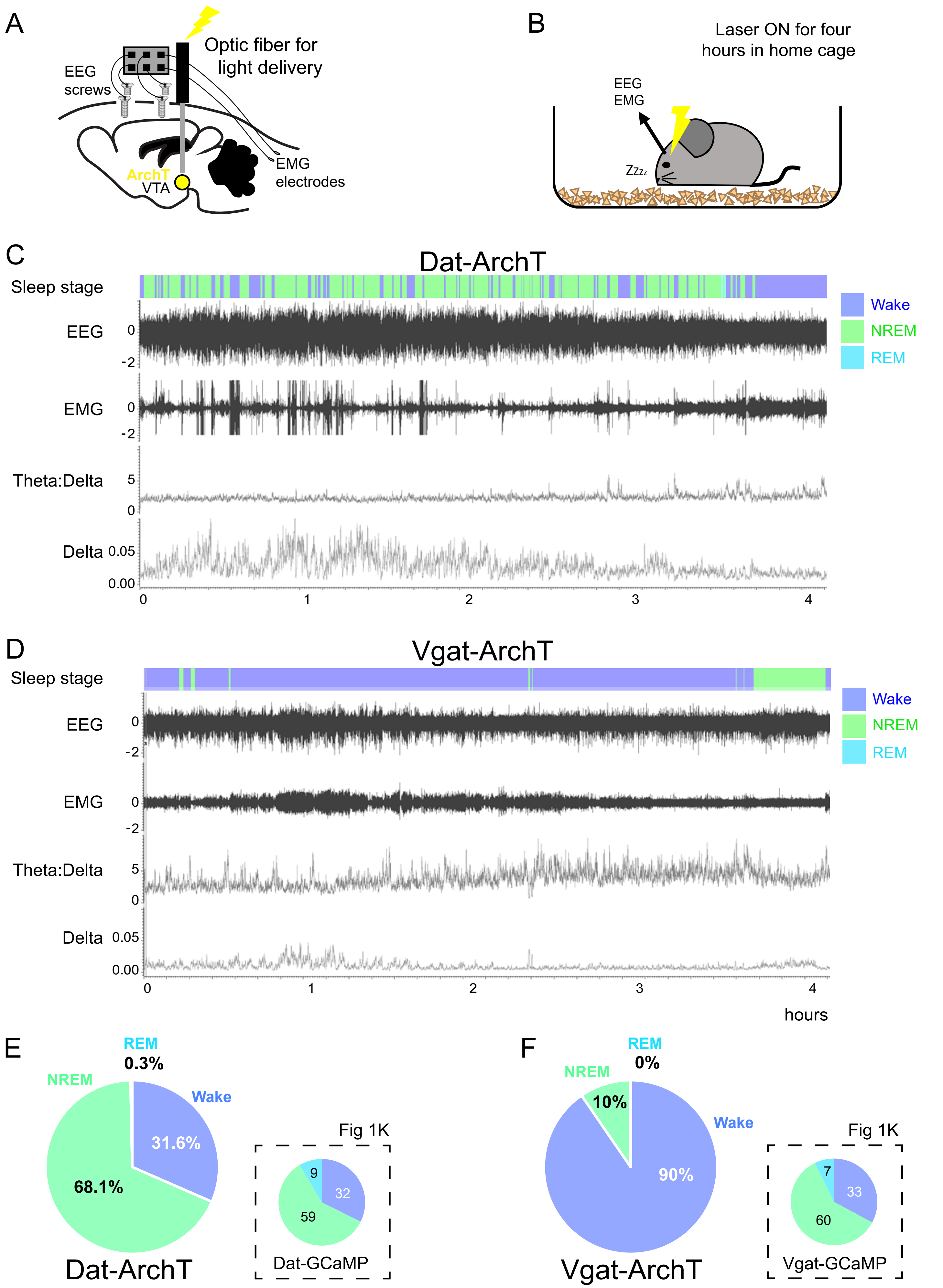

Supplementary Figure 5

Figure S5: Chronic opto-inhibition (related to Figures 3 and 4)

A. Experimental set-up for simultaneous sleep monitoring (using implanted EEG and EMG) and optogenetic inhibition (using implanted light fibre and cre-dependent ArchT).

B. In this chronic silencing experiment, the mouse was allowed to rest in its home cage while the laser was turned on continuously for four hours.

C. Raw four hour recording from chronic silencing of a Dat-ArchT mouse, showing EEG and EMG voltage, plus EEG Delta power and T:D ratio, which all inform sleep stage categorisation (top trace).

D. Raw four hour recording from chronic silencing of a Vgat-ArchT mouse, showing EEG and EMG voltage, plus EEG Delta power and T:D ratio, which all inform sleep stage categorisation (top trace - note the difference from C).

E. The percentage of time spent in different arousal states during chronic silencing of VTA Dat neurons (left), compared to the percentages when VTA Dat activity was passively monitored using photometry (right inset, from Figure 1).

F. The percentage of time spent in different arousal states during chronic silencing of VTA Vgat neurons (left), compared to the percentages when VTA Vgat activity was passively monitored using photometry (right inset, from Figure 1 - note difference from E).

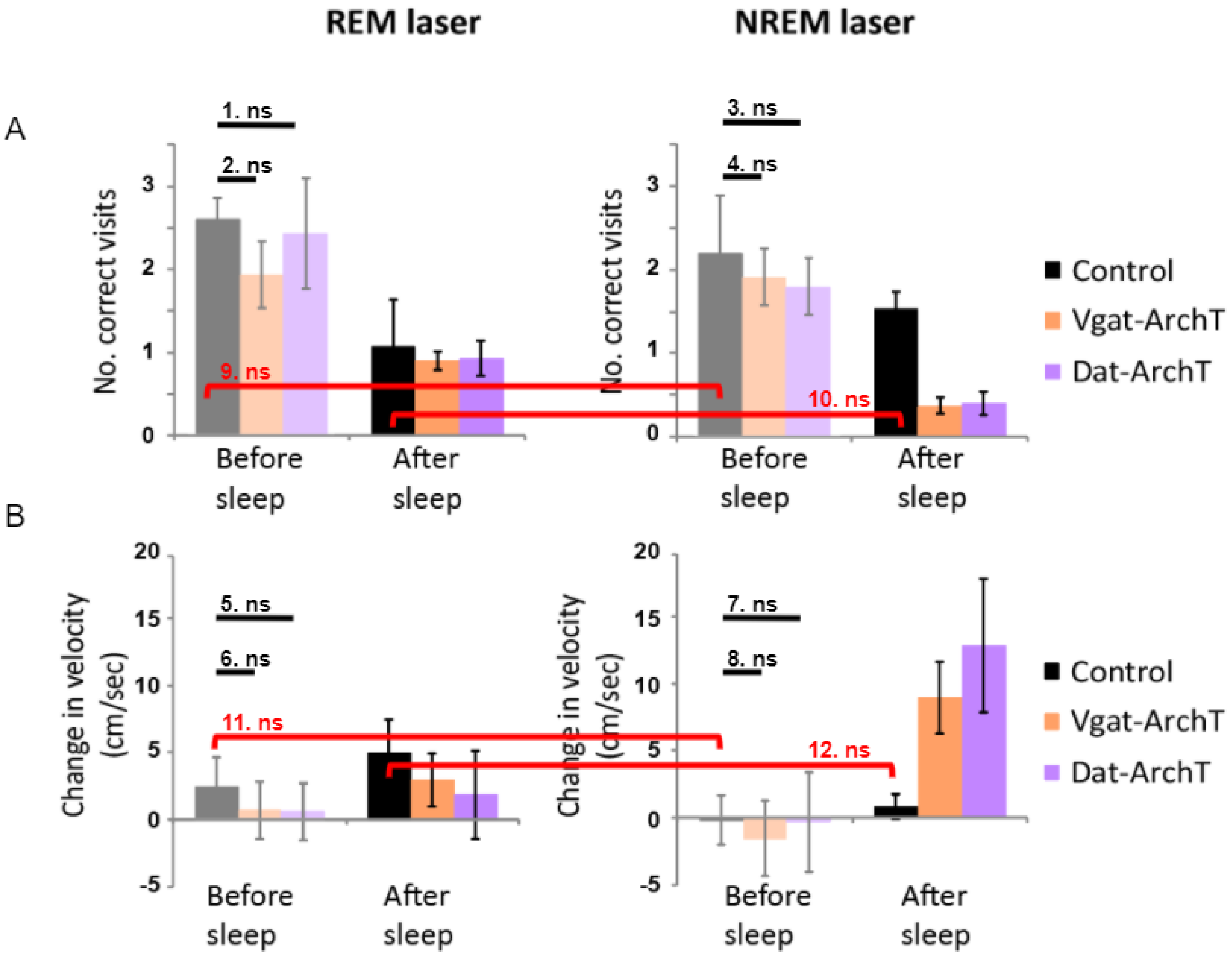

Supplementary Figure 6

Figure S6: Behavioural control comparisons (related to Figure 5)

There were no significant differences between control and experimental groups before manipulation (black comparisons), and control groups did not differ from each other before or after manipulation (red comparisons).

Black: Before sleep comparisons between groups (student's t-tests)

1,2 - Correct pod visits before sleep, REM: control vs Vgat (p=0.22); control vs dat (p=0.83)

3,4 - Correct pod visits before sleep, NREM: control vs Vgat (p=0.69); control vs dat (p=0.61)

5,6 - Velocity before sleep, REM: control vs Vgat (p=0.57); control vs Dat (p=0.54)

7,8 - Velocity before sleep, NREM: control vs Vgat (p=0.71); control vs Dat (p=0.97)

Red: Control group comparisons between laser conditions (student's t-tests)

9 - NREM vs REM correct pod visits in control group before sleep: no significant difference (p=0.59)

10 - NREM vs REM correct pod visits in control groups after sleep: no significant difference (p=0.46)

11 - NREM vs REM velocity in control group before sleep: no significant difference (p=0.37)

12 - NREM vs REM velocity in control group after sleep: no significant difference (p=0.16)

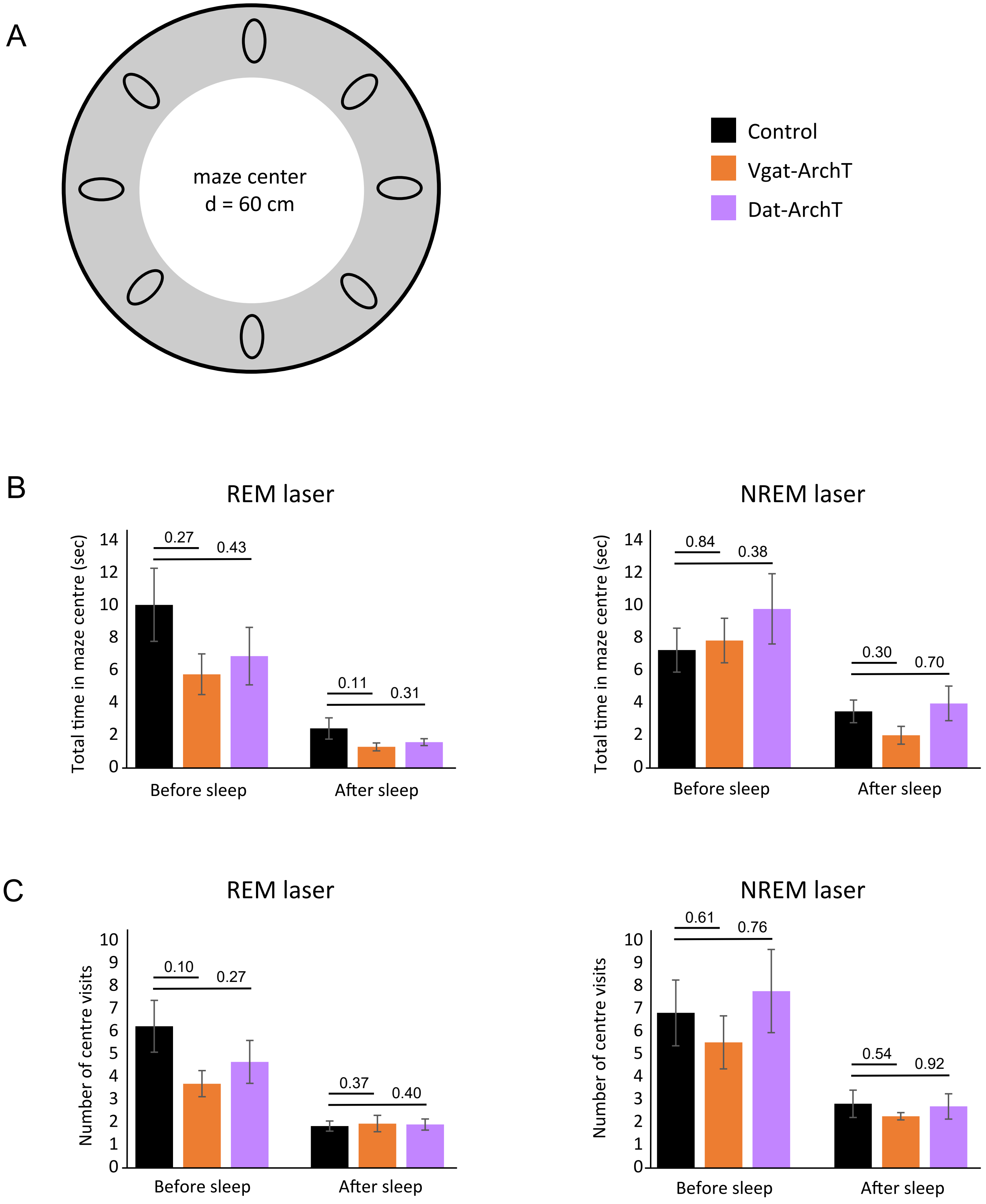

Supplementary Figure 7

Figure S7: Assessing anxiety in the maze (related to Figure 5)

A. For this analysis, the maze centre was defined as the circular region shown in white with 60 cm diameter, while the rest of the maze (shown in grey, with a width of 30 cm) was considered to be the "edge".

B. For both the REM laser condition (left; control n=5, Vgat n=6, Dat n=6) and NREM laser condition (right; control n=5, Vgat n=7, Dat n=5), the amount of time spent in the maze centre was not different between experimental and control groups, either before or after sleep (numbers above bar graphs represent p values from student's t-tests).

C. Similarly, the number of times that the mice entered the maze centre was not different between experimental and control groups either before or after sleep (tests and n numbers the same as in B).

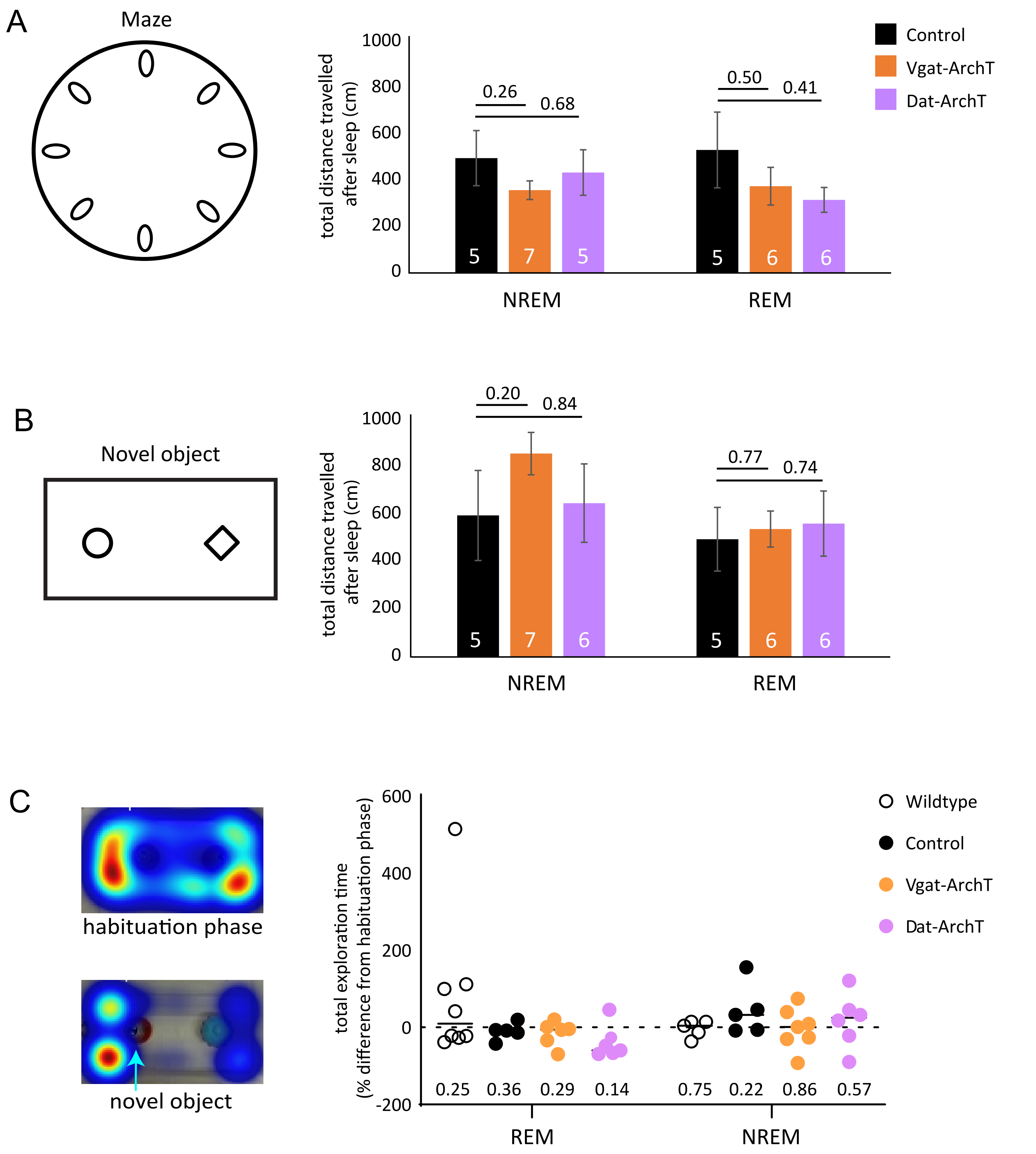

Supplementary Figure 8

Figure S8: Assessing general arousal (related to Figure 5)

A. For the maze experiment, the total distance that the mice travelled after sleep was not different between experimental and control groups, in either the NREM laser condition (left columns) or the REM laser condition (right columns). Numbers above bar graphs represent p values from student's t-tests; n numbers shown in white.

B. Similarly, for the novel object experiment, the total distance that the mice travelled after sleep was not different between experimental and control groups, in either the NREM laser condition (left columns) or the REM laser condition (right columns). Numbers above bar graphs represent p values from student's t-tests; n numbers shown in white.

C. There is no difference in the total exploration time between the non-opsin control mice and the wildtype mice (REM p=0.29; NREM p=0.17) or between the control group and the experimental groups for either REM manipulation (control vs Vgat p=0.77; control vs Dat p=0.20) or NREM manipulation (control vs Vgat p=0.20; GCaMP vs Dat p=0.54). Additionally, no group spent significantly more or less time exploring during the novel object phase than during the habituation phase (one sample t-tests do not reveal that any distribution is significantly different from zero, p values shown below each cluster).

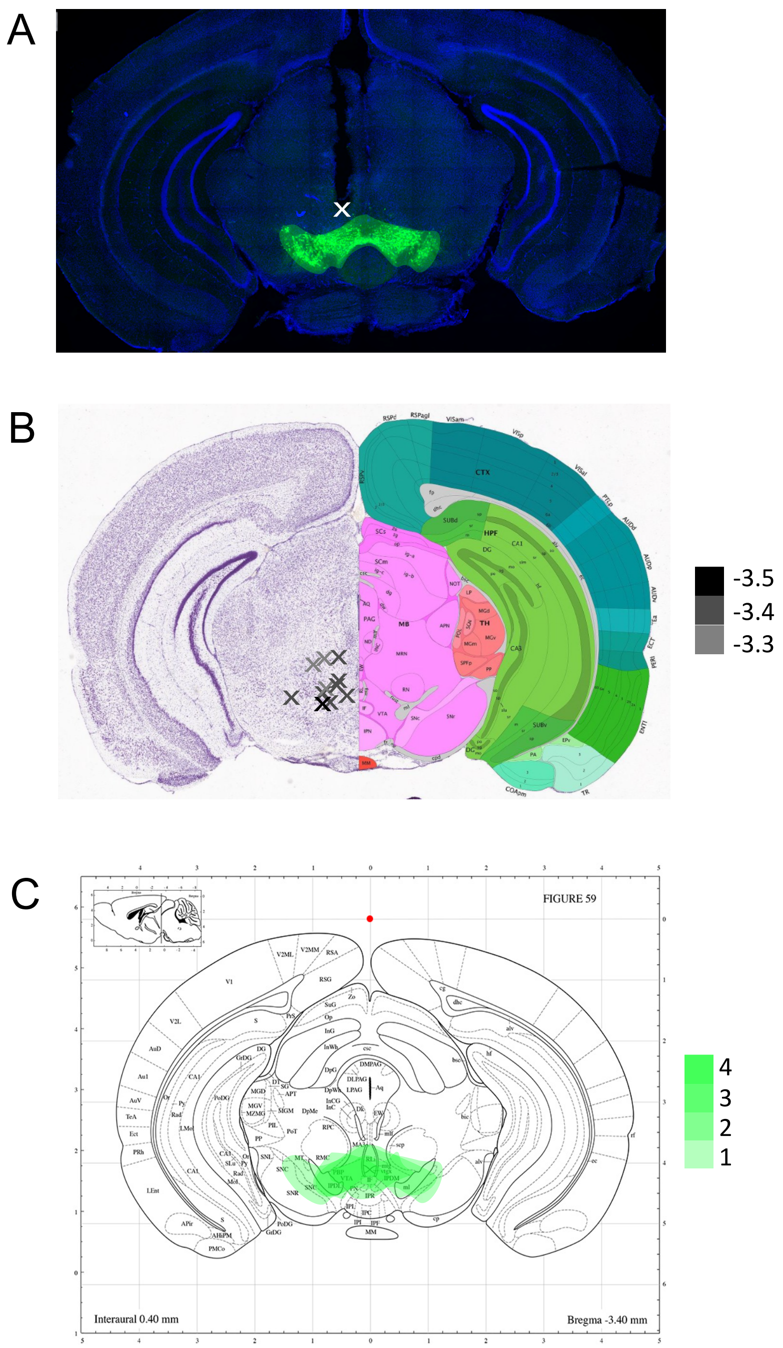

Supplementary Figure 9

Figure S9: Fiber placements and viral spread (related to Figures 1 and 3)

A. Example slice showing position of fiber tip (white x) and viral spread (transparent green shape).

B. Fiber tip positions from 11 brains (grey scale represents AP coordinate in which fiber tip was found).

C. Viral spread from four brains (colour scale represents the number of brains which showed expression in that region).
